# Tooth mineralization and histology patterns in extinct and extant snaggletooth sharks, *Hemipristis* (Carcharhiniformes, Hemigaleidae)—Evolutionary significance or ecological adaptation?

**DOI:** 10.1371/journal.pone.0200951

**Published:** 2018-08-08

**Authors:** Patrick L. Jambura, Cathrin Pfaff, Charlie J. Underwood, David J. Ward, Jürgen Kriwet

**Affiliations:** 1 Department of Palaeontology, University of Vienna, Vienna, Austria; 2 Department of Earth and Planetary Sciences, Birkbeck, University of London, London, United Kingdom; 3 Department of Earth Sciences, Natural History Museum, London, United Kingdom; Monash University, AUSTRALIA

## Abstract

Shark jaws exhibit teeth that are arranged into distinct series and files and display great diversities in shapes and structures, which not only is related to their function (grasping, cutting, crushing) during feeding, but also bear a strong phylogenetic signal. So far, most research on the relationship between shark teeth and feeding ecology and systematics focused on the external tooth morphology only. Although the tooth histology of sharks has been examined since the early 19^th^ century, its functional and systematic implications are still ambiguous. Shark teeth normally consist of either a porous, cellular dentine, osteodentine (in lamniform sharks and some batoids) or a dense layer of orthodentine (known from different sharks). Sharks of the order Carcharhiniformes, comprising ca. 60% of all extant shark species, are known to have orthodont teeth, with a single exception—the snaggletooth shark, *Hemipristis elongata*. High resolution micro-CT images of jaws and teeth from selected carcharhiniform sharks (including extant and fossil snaggletooth sharks) and tooth sections of teeth of *Hemipristis*, other carcharhiniform and lamniform sharks, have revealed that (1) *Hemipristis* is indeed the only carcharhiniform shark filling its pulp cavity with osteodentine in addition to orthodentine, (2) the tooth histology of *Hemipristis elongata* differs from the osteodont histotype, which evolved in lamniform sharks and conversely represents a modified orthodonty, and (3) this modified orthodonty was already present in extinct *Hemipristis* species but the mineralization sequence has changed over time. Our results clearly show the presence of a third tooth histotype—the pseudoosteodont histotype, which is present in *Hemipristis*. The unique tooth histology of lamniform sharks might provide a phylogenetic signal for this group, but more research is necessary to understand the phylogenetic importance of tooth histology in sharks in general.

## Introduction

Sharks possess a constantly forming series of teeth in which functional teeth are replaced in succession (polyphydont dentition) [1,2,3]. There are two main different patterns how teeth can be arranged within the jaw. They either are added alternatingly within two adjacent tooth series (double vertical row; alternate dentition) or in single tooth files (single vertical row; independent dentition) [4–6] with teeth being shed individually (e.g. in carcharhiniform sharks) [4,6] or in groups up to entire tooth rows (e.g. squaliform sharks) [4,7]. Teeth are initially formed within the dental lamina on the lingual side of the jaws and move toward the functional position on the jaw margin in a conveyor belt-like fashion [5,6,8]. The teeth on the jaw margin (situated labially) are in an erect position suitable for feeding, while those situated on the lingual face of the jaw cartilage are less developed and are inverted (with the tip of the tooth directed lingually towards the dental lamina) or in a semi-erect position. The number of teeth in an erect position within each tooth file varies between species. These erect teeth are the ones in use and thus are considered functional teeth, teeth in a semi-erect or inverted position form the developing replacement teeth [4].

Shark teeth show a high diversity of different morphologies that are thought to be related to different trophic adaptations, i.e. grasping, cutting, or crushing the prey [2, 3,9,10]. Recent works on biomechanics indicate that this link might exist, but evidence for it is cloudy at best to date [11–14]. Shark tooth morphology has been extensively investigated and is known to bear strong taxonomic and systematic signals, with descriptions of fossil shark species mostly based on isolated teeth [15–18]. Despite extensive study on shark teeth, the impact of the histological tooth composition on feeding mechanics or phylogeny is largely unknown.

The tooth crowns in elasmobranchs consist of two material zones, the outer enameloid (which is structurally similar to, but not homologous with, the enamel found in osteichthyans (including tetrapods) [19,20]) and a central core of dentine [21–25]). In elasmobranchs, two different tooth histotypes can be distinguished according to the mineralization pattern of the central tooth crown dentine [1,22,23,25]. Orthodont teeth have hollow pulp cavities surrounded by orthodentine underling the enameloid, a dental structure known from most sharks and rays [23,25–32]). In contrast, osteodont teeth have the pulp cavity filled completely with osteodentine, a dental structure mainly known in lamniform sharks [24,25,33] and some batoids [31,34]. These different histotypes were assumed to bear a phylogenetic signal [22,35], but this is challenged by data on hybodont sharks, the sister group to modern sharks and rays [36,37], which revealed the presence of both histotypes within hybodont sharks of the same genus [38]. Within the shark and ray crown group, osteodonty is known from a relatively small number of shark taxa, but these are present across several different shark clades [23–25,28,39] as well as within rays of the order Myliobatiformes [31,34]. However, the reason for the presence of two different tooth histologies in sharks and rays is uncertain, as histotypes cross both large scale taxonomic groups and tooth morphotypes.

The snaggletooth shark *Hemipristis elongata* (Hemigaleidae, Carcharhiniformes) is the sole extant species of its genus, and the only carcharhiniform shark proposed to have the osteodont tooth histology [2,23,39]. All other carcharhiniforms that have been studied so far are characterized by orthodont teeth. By examining and comparing the tooth mineralization process of *Hemipristis elongata* with other carcharhiniform sharks, using microCT-scanning, as well as examining the tooth histology of the extinct species, †*Hemipristis serra* (from the Miocene) and †*Hemipristis curvatus* (from the Eocene) we intend to gain new information about the tooth histology in *Hemipristis*. By comparing the tooth mineralization of *Hemipristis elongata* with lamniform sharks we ultimately try to resolve the question, if they actually share the same tooth histology, or if a comparable tooth histology evolved independently in *Hemipristis*.

## Material and methods

For this study, a total of 12 dried jaws and 20 teeth of extant and extinct shark species were examined. Each jaw and a total of 19 teeth were scanned using a SkyScan1173 microCT device (Bruker, Kontich, Belgium). Settings for each specimen examined here are provided in the Supporting Information section (S1 Table). Additional, sections of isolated teeth were prepared to elucidate the histology using a KEYENCE 3D Digital VHX-600 microscope.

Extinct forms are denoted with preceding daggers. Five jaws analysed here are from hemigaleid sharks (two *Hemipristis elongata*, one each of *Chaenogaleus macrostoma*, *Hemigaleus microstoma*, *Paragaleus randalli*), six jaws are from carcharhinid sharks (*Carcharhinus melanopterus*, *C*. *obscurus*, *C*. *signatus*, *Galeocerdo cuvier*, *Prionace glauca*, *Rhizoprionodon acutus*) and one jaw is from a sphyrnid shark (*Sphyrna zygaena*) (Table 1).

**Table 1:** Summary of all examined shark jaws. Specimens are either deposited in the collection of the Department of Palaentology, University of Vienna (UV) or in the collection of the Earth and Planetary Sciences of Birkbeck, University of London (BBK) and are publicly accessible.

Species	Family	Accession number	Museum/Collection	Locality
*Carcharhinus melanopterus*	Carcharhinidae	EMRG-Chond-J-3	UV	Haus des Meeres, Austria
*Carcharhinus obscurus*	Carcharhinidae	EMRG-Chond-J-5	UV	Florida, USA
*Carcharhinus signatus*	Carcharhinidae	EMRG-Chond-J-4	UV	Florida, USA
*Galeocerdo cuvier*	Carcharhinidae	EMRG-Chond-J-13	UV	Taiwan
*Prionace glauca*	Carcharhinidae	EMRG-Chond-J-6	UV	Portugal
*Rhizoprionodon acutus*	Carcharhinidae	EMRG-Chond-J-7	UV	Angola
*Chaenogaleus macrostoma*	Hemigaleidae	CD042	BBK	Kuwait
*Hemigaleus microstoma*	Hemigaleidae	CD045	BBK	Taiwan
*Hemipristis elongata*	Hemigaleidae	EMRG-Chond-J-1	UV	Sri Lanka
*Hemipristis elongata*	Hemigaleidae	EMRG-Chond-J-2	UV	Java
*Paragaleus randalli*	Hemigaleidae	CD046	BBK	Kuwait
*Sphyrna zygaena*	Sphyrnidae	EMRG-Chond-J-8	UV	Japan

We examined 20 teeth, 17 were from extinct *Hemipristis* species, of which seven were from the Miocene (†*Hemipristis serra*) and ten from the Eocene (†*Hemipristis curvatus*). Two orthodont teeth of extant species, the tiger shark *Galeocerdo cuvier* and the bull shark *Carcharhinus leucas*, were also scanned. Additionally, three teeth of †*Hemipristis serra*, *Carcharhinus leucas*, and the shortfin mako *Isurus oxyrinchus* (Lamniformes) were sectioned horizontally and tooth histology was examined under the light microscope (Table 2).

**Table 2:** Summary of all examined extant and fossil shark teeth. All specimens are deposited in the collection of the Department of Palaentology at the University of Vienna and are publicly accessible.

Species	Family	Accession number	Age	Locality
*Carcharhinus leucas*	Carcharhinidae	EMRG-Chond-T-15	extant	Brisbane, Australia
*Galeocerdo cuvier*	Carcharhinidae	EMRG-Chond-T-16	extant	Jakarta, Indonesia
†*Hemipristis curvatus*	Hemigaleidae	EMRG-Chond-T-17	34–38 Mya	Samlat Formation, Ad-Dakhla, Morocco
†*Hemipristis curvatus*	Hemigaleidae	EMRG-Chond-T-18	34–38 Mya	Samlat Formation, Ad-Dakhla, Morocco
†*Hemipristis curvatus*	Hemigaleidae	EMRG-Chond-T-19	34–38 Mya	Samlat Formation, Ad-Dakhla, Morocco
†*Hemipristis curvatus*	Hemigaleidae	EMRG-Chond-T-20	34–38 Mya	Samlat Formation, Ad-Dakhla, Morocco
†*Hemipristis curvatus*	Hemigaleidae	EMRG-Chond-T-21	34–38 Mya	Samlat Formation, Ad-Dakhla, Morocco
†*Hemipristis curvatus*	Hemigaleidae	EMRG-Chond-T-22	34–38 Mya	Samlat Formation, Ad-Dakhla, Morocco
†*Hemipristis curvatus*	Hemigaleidae	EMRG-Chond-T-32	34–38 Mya	Samlat Formation, Ad-Dakhla, Morocco
†*Hemipristis curvatus*	Hemigaleidae	EMRG-Chond-T-33	34–38 Mya	Samlat Formation, Ad-Dakhla, Morocco
†*Hemipristis curvatus*	Hemigaleidae	EMRG-Chond-T-34	34–38 Mya	Samlat Formation, Ad-Dakhla, Morocco
†*Hemipristis curvatus*	Hemigaleidae	EMRG-Chond-T-35	34–38 Mya	Samlat Formation, Ad-Dakhla, Morocco
†*Hemipristis serra*	Hemigaleidae	EMRG-Chond-T-9	15–19 Mya	Pungo River Formation, North Carolina
†*Hemipristis serra*	Hemigaleidae	EMRG-Chond-T-10	15–19 Mya	Pungo River Formation, North Carolina
†*Hemipristis serra*	Hemigaleidae	EMRG-Chond-T-11	15–19 Mya	Pungo River Formation, North Carolina
†*Hemipristis serra*	Hemigaleidae	EMRG-Chond-T-12	15–19 Mya	Pungo River Formation, North Carolina
†*Hemipristis serra*	Hemigaleidae	EMRG-Chond-T-29	15–19 Mya	Pungo River Formation, North Carolina
†*Hemipristis serra*	Hemigaleidae	EMRG-Chond-T-30	15–19 Mya	Pungo River Formation, North Carolina
†*Hemipristis serra*	Hemigaleidae	EMRG-Chond-T-31	15–19 Mya	Pungo River Formation, North Carolina
*Isurus oxyrinchus*	Lamnidae	EMRG-Chond-T-14	extant	unknown

The obtained micro-CT data sets of the jaws and teeth were loaded into the software package DataViewer (version 1.5.1.2 (64bit), SkyScan (Bruker micro-CT, Kontich, Belgium) and Amira software packages (version 5.4.5, FEI Visualization Sciences Group, Oregon, USA). This resulted in 2D pictures of virtual sections through jaws and teeth, displaying the degree of mineralization in each tooth within any given tooth file. A tooth file consists of the most labially situated tooth and all following replacement teeth, displaying all developmental stages [24]. The resulting 2D images were edited using Adobe Photoshop CS6 (version 13.0, Adobe Systems, San Jossé, USA) concerning colour balance, contrast and labelling.

**Fig 1:**
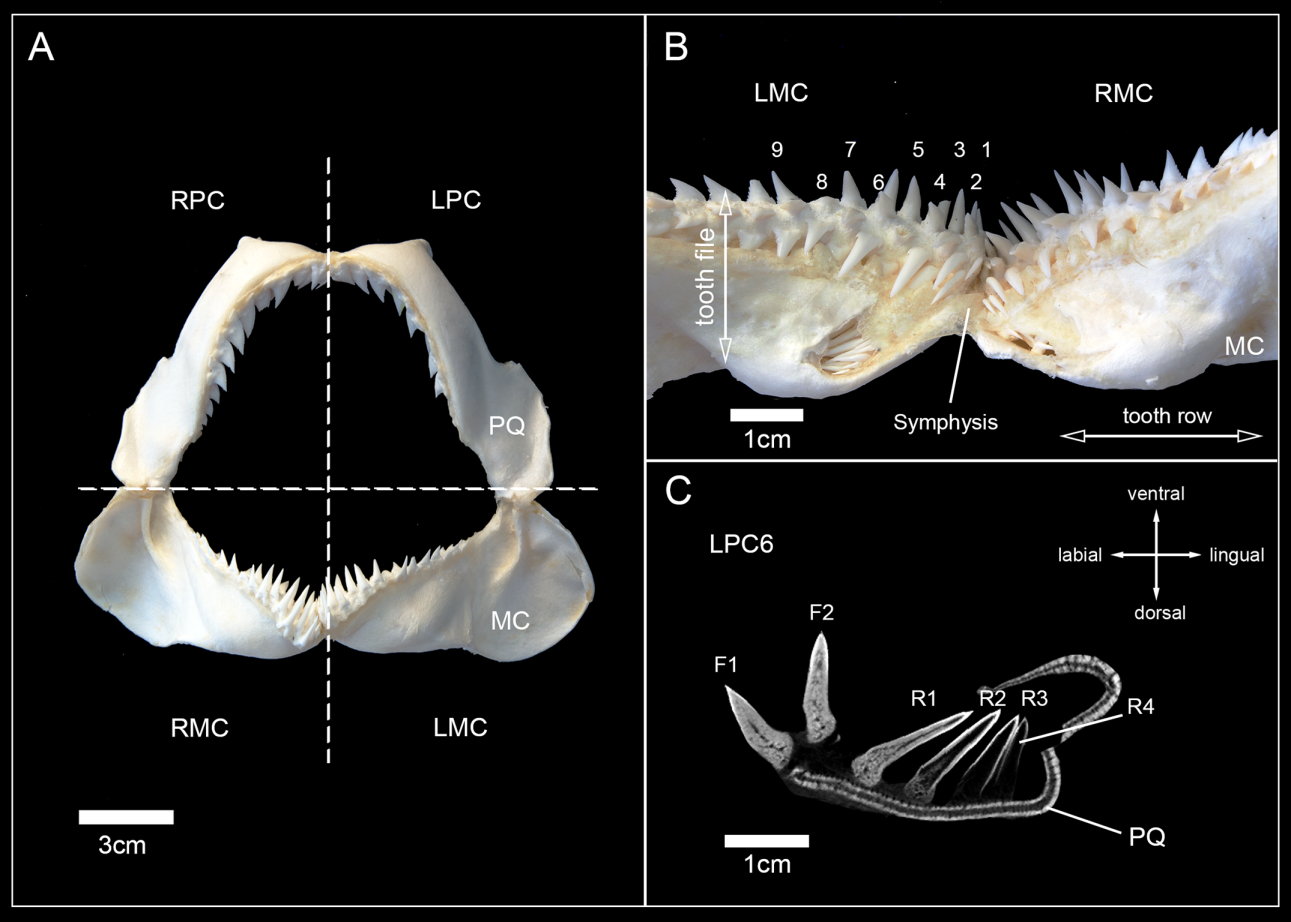
Terminology used to describe the position of teeth within the jaw and tooth files. (A) Jaw of *Hemipristis elongata* in anterior view, (B) anterior part of the lower jaw of *Hemipristis elongata*, (C) Tooth series LPC6 of *Hemipristis elongata*. F, functional tooth; LMC, left Meckel’s cartilage; LPC, left palatoquadrate cartilage; MC, Meckel’s cartilage (lower jaw); PQ, palatoquadrate cartilage (upper jaw); R, replacement tooth; RMC, right Meckel’s cartilage, RPC, right palatoquadrate cartilage.

Terminology for tooth positions was adapted from the literature [33], with teeth being termed as follows: The first letter indicates the location of the tooth file within left or right jaws, respectively (L, R). The next two letters are either MC (Meckel’s cartilage; lower jaw) or PC (palatoquadrate cartilage; upper jaw), followed by a number, which determines the position of the file distally to the symphysis (MC1 = next to the symphysis, MC2 = second file next to the symphysis, etc.). Functional teeth (F) and replacement teeth (R) additionally were distinguished. A functional tooth was defined as being fully mineralized and in an erect or semi-erect position, which allows it to be utilized for food gathering (e.g. grabbing, cutting, impaling, etc.). Replacement teeth are located lingually to the functional tooth or teeth and are not yet fully developed at this point. Their apex usually points lingually. The position of teeth within the tooth file is numbered in regard to their position (functional or replacement) (Fig 1).

## Results

### Tooth development and mineralization in carcharhiniform sharks

High-resolution micro-CT images of tooth files from selected carcharhiniform sharks of the families Carcharhinidae, Hemigaleidae and Sphyrnidae were used to gain a better understanding of tooth development and tooth mineralization processes in this group. For this, we examined the anterior tooth files, in *Hemipristis elongata* also lateral and posterior tooth files, to obtain very detailed information throughout the dentition. Tooth development and mineralization were consistent through all tooth files of a species, and independent of the position of the tooth file within the jaw.

**Fig 2:**
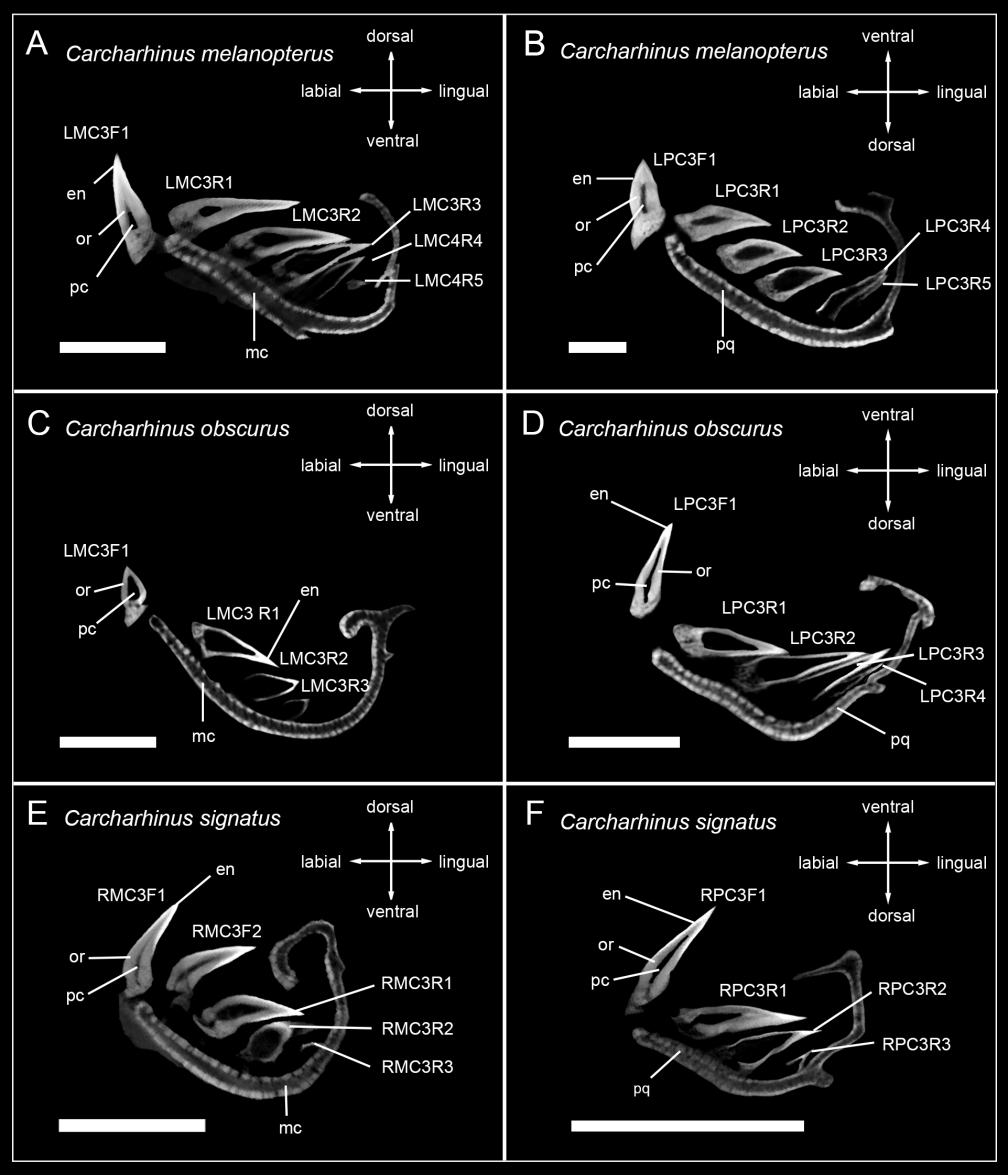
Micro-CT images of tooth series of sharks of the genus *Carcharhinus*. Tooth files showing the number of teeth, tooth stages and mineralization in (A) LMC3 (lower jaw) *C*. *melanopterus*, (B) LPC3 (upper jaw) *C*. *melanopterus* (EMRG-Chond-J-3), (C) LMC3 *C*. *obscurus*, (D) LPC3 *C*. *obscurus* (EMRG-Chond-J-5), (E) RMC3 *C*. *signatus*, (F) RPC3 *C*. *signatus* (EMRG-Chond-J-4). Scalebar = 0.5cm. en, enameloid; mc, Meckel’s cartilage; or, orthodentine; pc, pulp cavity; pq, palatoquadrate.

**Fig 3:**
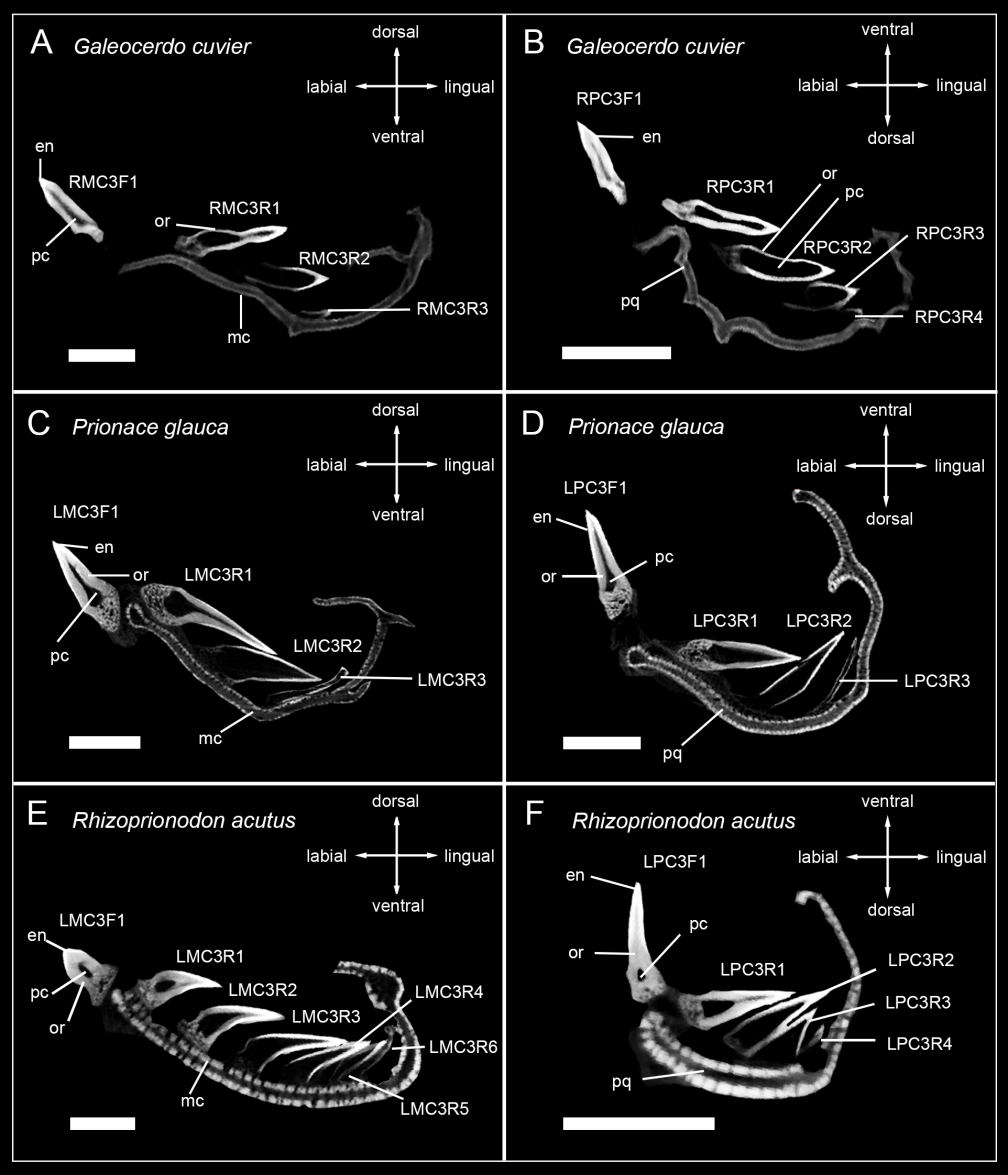
Micro-CT images of tooth series of the carcharhinid sharks *Galeocerdo cuvier*, *Prionace glauca*, and *Rhizoprionodon acutus*. Tooth files showing the number of teeth, tooth stages and mineralization in (A) RMC3 (lower jaw) *Galeocerdo cuvier*, (B) RPC3 (upper jaw) *Galeocerdo cuvier* (EMRG-Chond-J-13), (C) LMC3 *Prionace glauca*, (D) LPC3 *Prionace glauca* (EMRG-Chond-J-6), (E) LMC3 *Rhizoprionodon acutus*, (F) LPC3 *Rhizoprionodon acutus* (EMRG-Chond-J-7). Scalebar = 0.5cm. en, enameloid; mc, Meckel’s cartilage; or, orthodentine; pc, pulp cavity; pq, palatoquadrate.

**Fig 4:**
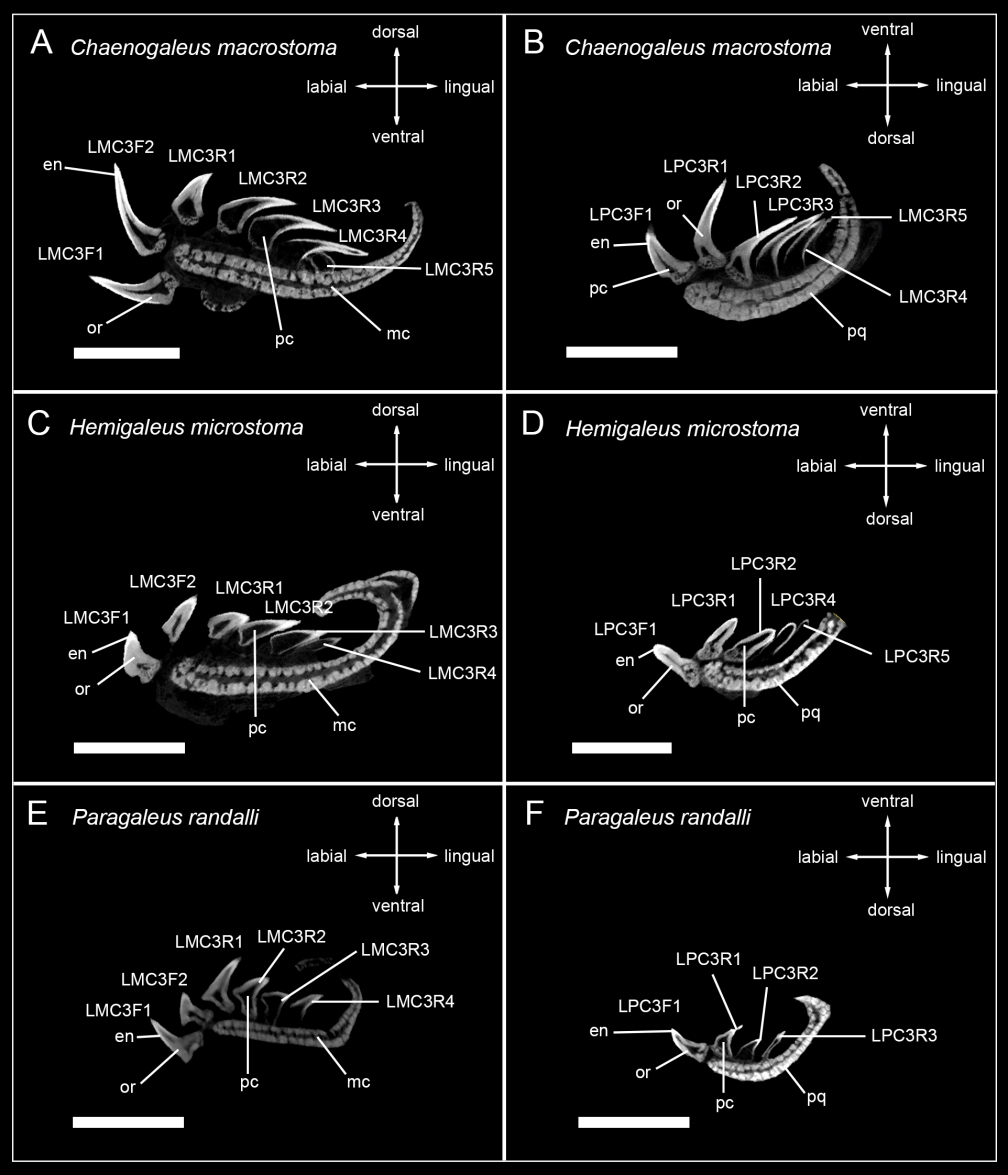
Micro-CT images of tooth series of sharks of the family Hemigaleidae. Tooth files showing the number of teeth, tooth stages and mineralization in (A) LMC3 (lower jaw) *Chaenogaleus macrostoma*, (B) LPC3 (upper jaw) *Chaenogaleus macrostoma* (CD042), (C) LMC3 *Hemigaleus microstoma*, (D) LPC3 *Hemigaleus microstoma* (CD045), (E) LMC3 *Paragaleus randalli*, (F) LPC3 *Paragaleus randalli* (CD046). Scalebar = 0.5cm. en, enameloid; mc, Meckel’s cartilage; or, orthodentine; pc, pulp cavity; pq, palatoquadrate.

**Fig 5:**
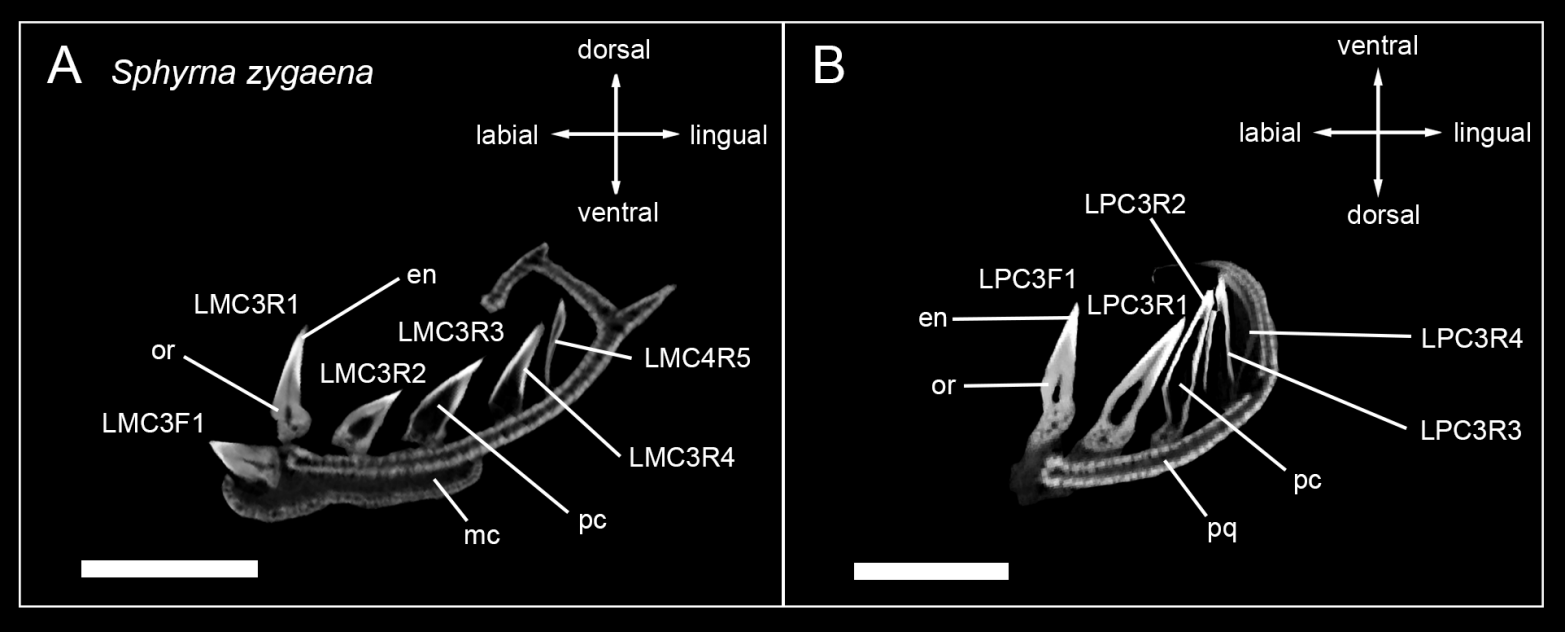
Micro-CT images of tooth series of the sphyrnid shark *Sphyrna zygaena*. Tooth files showing the number of teeth, tooth stages and mineralization. (A) LMC3 (lower jaw), (B) LPC3 (upper jaw) of *Sphyrna zygaena* (EMRG-Chond-J-8). Scalebar = 0.5cm. en, enameloid; mc, Meckel’s cartilage; or, orthodentine; pc, pulp cavity; pq, palatoquadrate.

The tooth mineralization sequence is consistent in all observed carcharhiniform species except in *Hemipristis elongata*, with variations due to different numbers of teeth within tooth files of different species. The structure that always mineralizes first is the tooth enameloid. It starts to mineralize in the apex of the crown and is detectable only as a very thin layer before it is becoming more prominent and extending basally over the whole tooth crown surface during anterior progression of the tooth within the file. After the enameloid is formed, the formation of the root starts either simultaneously with the emergence of orthodentine in the crown or slightly before that and is only detectable as a very light spongy structure. As the tooth further develops, the orthodentine layer in the crown becomes thicker and the osteodentine within the root becomes denser. In fully formed and functional teeth, the hollow pulp cavity is maintained and therefore clearly reflects the orthodont histotype (Figs 2–5).

### Tooth mineralization in the snaggletooth shark *Hemipristis elongata*

**Fig 6:**
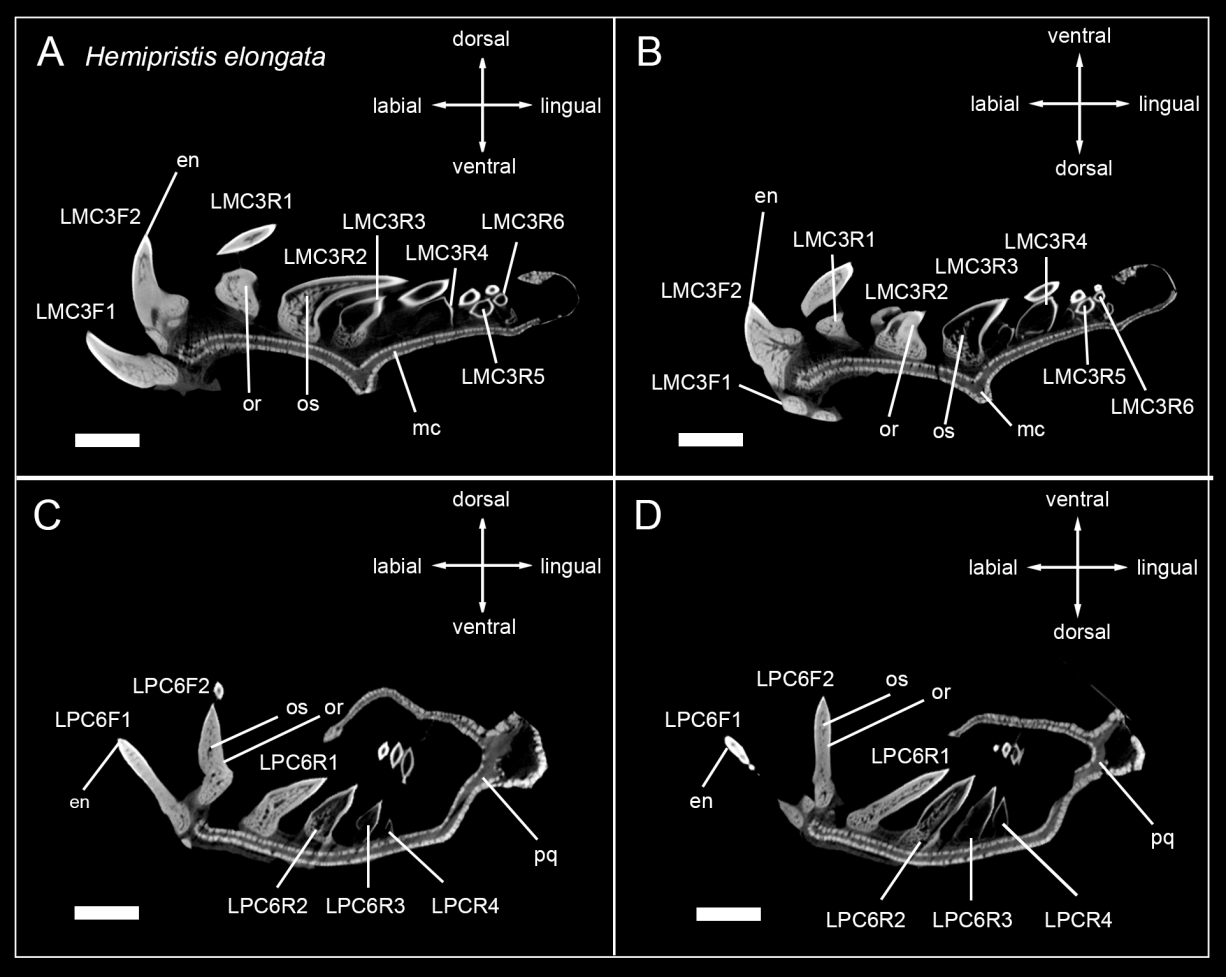
Micro-CT images of tooth series of *Hemipristis elongata*. Tooth files showing the number of teeth, tooth stages and mineralization in (A), (B) LMC3 (lower jaw), (C), (D) LPC6 (upper jaw) of *Hemipristis elongata* EMRG-Chond-J-1. Scalebar = 0.5cm. en, enameloid; mc, Meckel’s cartilage; or, orthodentine; os, osteodentine; pc, pulp cavity; pq, palatoquadrate.

**Fig 7:**
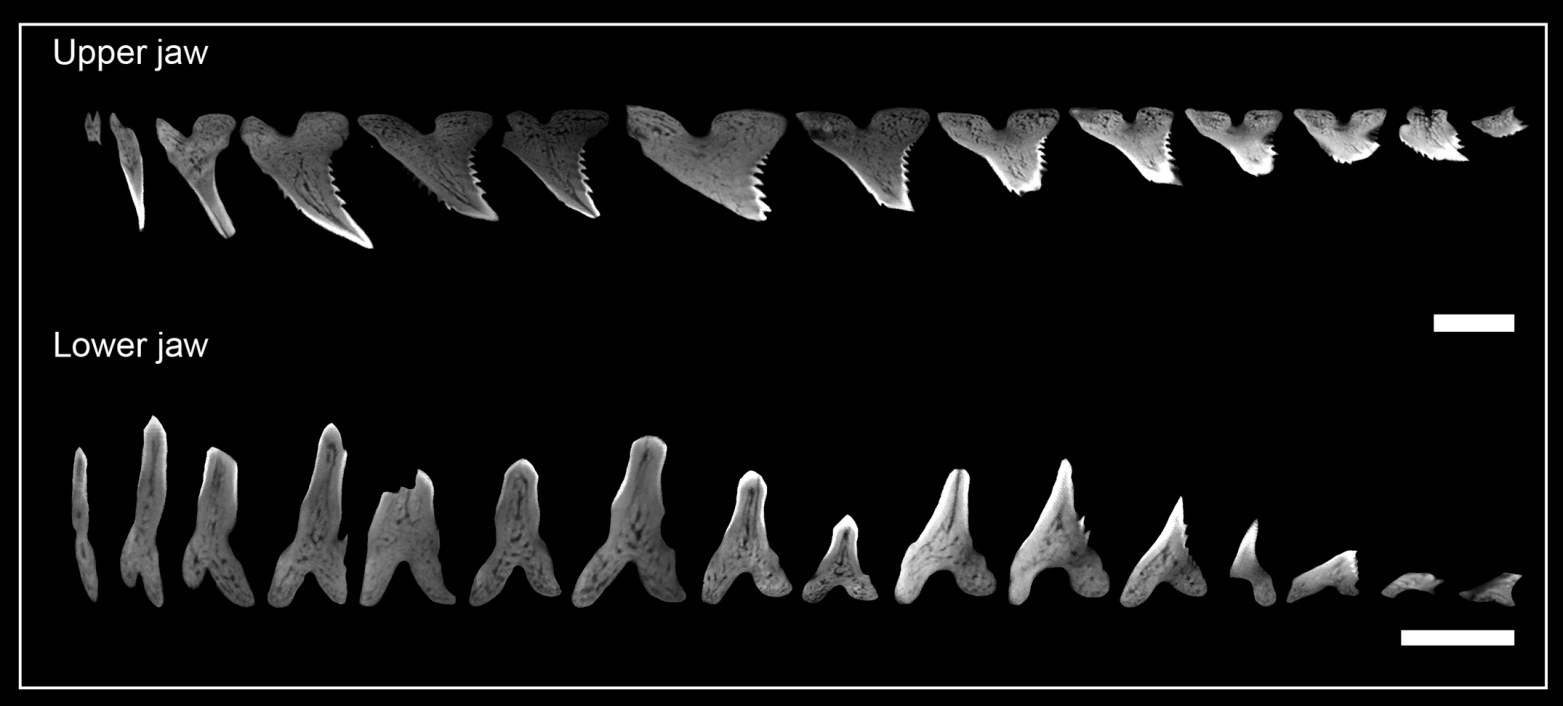
Micro-CT images of functional teeth of each tooth series in the lower and upper jaw of Hemipristis elongata. The jaws show strong dignathic and monognathic heterodonty with slender anterior teeth for grasping, and serrated lateral teeth for cutting the prey. Although the morphology of teeth within a horizontal row change drastically, the tooth histology is the same for each functional tooth. Scalebar = 0.5cm.

The initial tooth mineralization sequence in *Hemipristis elongata* is very similar to other carcharhiniform sharks, but very shortly after the formation of the enameloid the pulp cavity starts to fill with osteodentine, until the pulp cavity is replaced completely by an osteodentine core. In adjacent tooth files of the lower jaw we found that teeth, which occupy the same position within their respective tooth files, differ slightly in their degree of mineralization. To illustrate this, the third and fourth tooth file left of the symphysis in the lower and upper jaws are used to describe the tooth development in *Hemipristis elongata*. The most lingual teeth (LMC3R6, LMC4R6, LPC3R4, LPC4R5), representing the earliest stage of mineralization, lack any ortho- or osteodentine and the only mineralized structure present is enameloid. Enameloid first starts forming in the apex of the crown and successively extends basally until it covers the whole crown. The mineralization of the enameloid is completed very early during tooth development and serrations at the crown are already formed (LMC3R4, LMC4R4, LPC3R3, LPC4R4). After completion of the enameloid, the root starts to mineralize in LMC4R4. Orthodentine and osteodentine are still lacking in the crown. In LMC3R3, LPC3R2, and LPC4R2, the root starts to mineralize simultaneously with the formation of a thin orthodentine layer below the enameloid. Osteodentine is already reaching into the pulp cavity of the crown from below as an extension of the root osteodentine (LMC3R3, LPC3R2, LPC4R3). During the next steps of tooth development, the orthodentine layer increases in thickness medially and osteodentine expands apically into the pulp cavity, until the pulp cavity is fully filled (LMC3F2, LMC4R1, LPC3F2, LPC4F1) (Fig 6 and Table 3). Although the degree of mineralization might differ slightly in adjacent tooth files, all tooth files follow the same mineralization pattern and functional teeth always have filled pulp cavities (Fig 7).

**Table 3:** Mineralization sequence in *Hemipristis elongata*. Table showing the degree of mineralization for every tooth within the tooth files LMC3 and LMC4. Tooth files of the lower jaw show an alternate mineralization pattern, with replacement teeth of one file showing a higher degree of mineralization than teeth of the adjacent file. en, enameloid; or, orthodentine; os, osteodentine.

*Hemipristis elongata*									
LMC3					LMC4				
Tooth	en	or	os	Root	Tooth	En	or	os	Root
F1	Complete	Complete	Fully mineralized	Fully mineralized	F1	Complete	Complete	Fully mineralized	Fully mineralized
F2	Complete	Complete	Fully mineralized	Fully mineralized	R1	Complete	Complete	Fully mineralized	Fully mineralized
R1	Complete	Incomplete	Cavity almost fully filled	Almost fully mineralized	R2	Complete	Incomplete	Cavity partially filled	Partially mineralized
R2	Complete	Incomplete	Cavity partially filled	Partially mineralized	R3	Complete	Incomplete	Cavity partially filled	Partially mineralized
R3	Complete	Incomplete	Start of osteodentine formation	Root formation	R4	Complete	Absent	Hollow	Root formation
R4	Complete	Absent	Hollow	Absent	R5	Incomplete	Absent	Hollow	Absent
R5	Incomplete	Absent	Hollow	Absent	R6	Incomplete	Absent	Hollow	Absent
R6	Incomplete	Absent	Hollow	Absent	-	-	-	-	-

### Tooth mineralization in †*Hemipristis serra* and †*Hemipristis curvatus*

With the new knowledge about the tooth mineralization sequence in *Hemipristis elongata*, the next step was to look at fossil *Hemipristis* species to get a better understanding about the origin and evolution of the unique tooth mineralization pattern in *Hemipristis elongata* within carcharhiniform sharks. For this, isolated fossil teeth of the two extinct species †*Hemipristis serra* (from the Miocene) and †*Hemipristis curvatus* (from the Eocene), the latter one being the oldest known *Hemipristis* species were analysed using micro-CT images.

**Fig 8:**
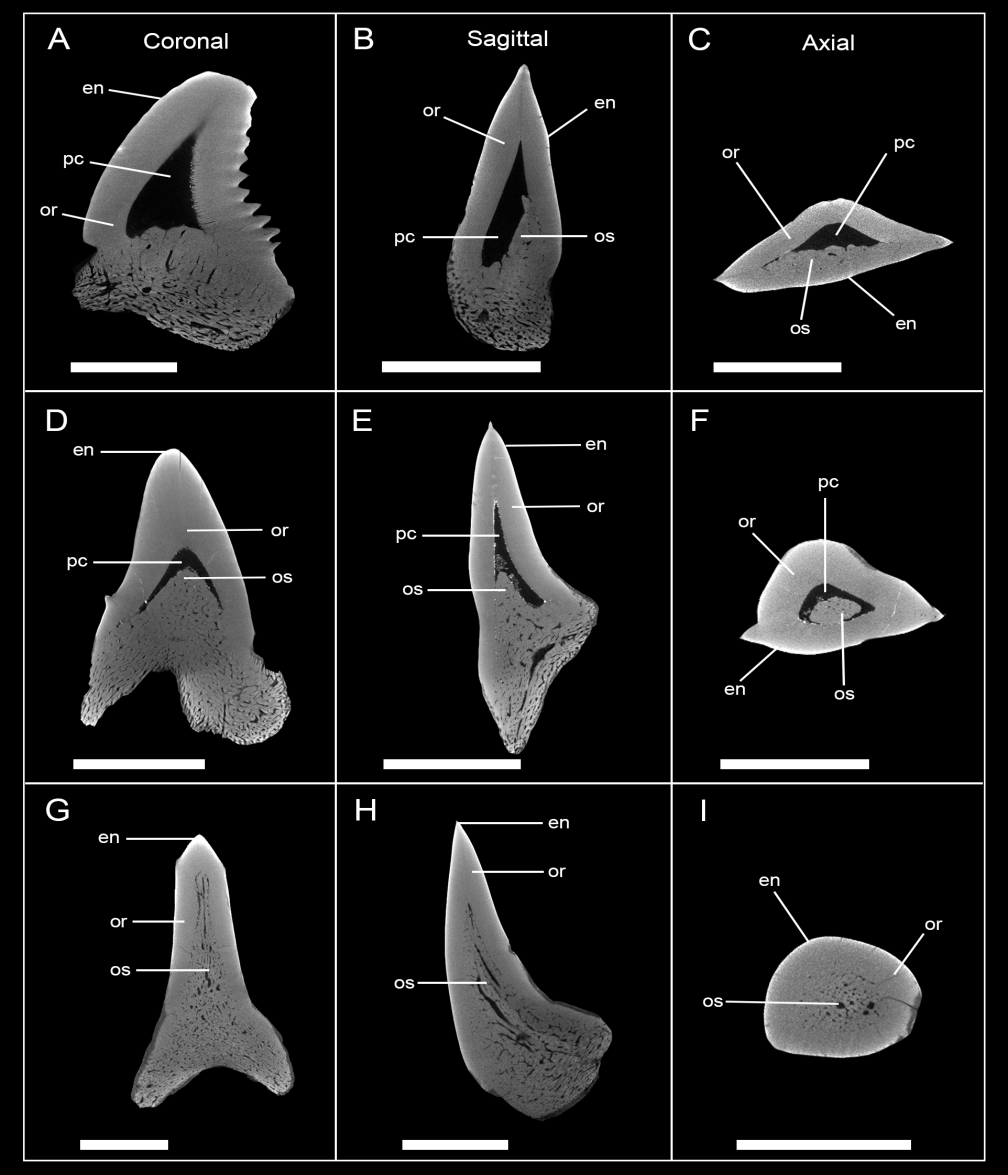
Micro-CT images of tooth sections of different developmental stages in †*Hemipristis serra* in frontal, sagittal and axial view. (A), (B), (C) EMRG-Chond-T-9, Pulp cavity hollow with little intrusion of osteodentine; (D), (E), (F) EMRG-Chond-T-12, Pulp cavity hollow, half filled with osteodentine; (G), (H), (I) EMRG-Chond-T-10, pulp cavity filled, tooth fully mineralized; scalebar = 1cm; en, enameloid; mc, Meckel’s cartilage; or, orthodentine; os, osteodentine; pc, pulp cavity; pq, palatoquadrate.

**Fig 9:**
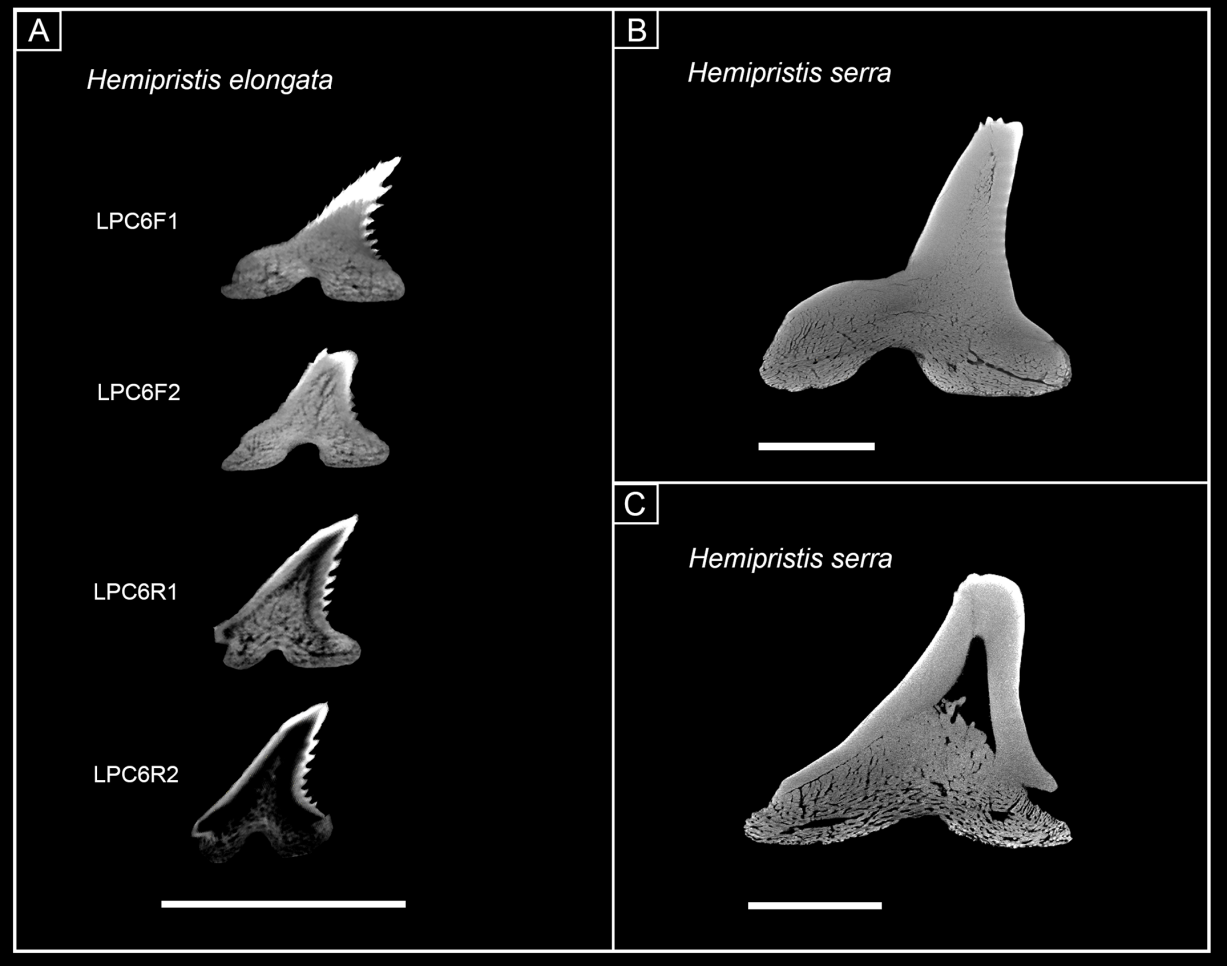
Root mineralization in *Hemipristis*. (A) Mineralization sequence of the root in *Hemipristis elongata*; (B) fully mineralized tooth with fully filled pulp cavity and dense osteodentine in the root in †*Hemipristis serra* (EMRG-Chond-T-11); (C) tooth with hollow pulp cavity in the crown and porous osteodentine within the root displaying a replacement tooth of †*Hemipristis serra* (EMRG-Chond-T-9). Scalebar = 1cm.

The examined teeth of †*Hemipristis serra* were from different positions within the jaw: five teeth represent upper laterals, one an upper anterior and one a lower anterior tooth (Fig 8, S1 and S2 Figs). CT-images revealed different developmental stages in the upper lateral teeth. Specimens EMRG-Chond-T-9 and EMRG-Chond-T-31 represent the earliest developmental stages of the examined teeth. They already show mineralization of the root and a thick and prominent layer of orthodentine under a layer of enameloid, but the pulp cavity still is unfilled (Fig 8A–8C, S1 and S2 Figs). In sagittal view, osteodentine is detectable as a protrusion of the upper edge of the root osteodentine, entering the pulp cavity basally (Fig 8B, S1 and S2 Figs). The roots are fully formed but are very porous and not as mineralized as the roots of teeth with fully filled pulp cavities, additionally demonstrating the earlier stage of development (Fig 9). The upper lateral tooth EMRG-Chond-T-31 shows higher degree of osteodentine intrusion from the root into the pulp cavity, but a (smaller) hollow pulp cavity is maintained (S2 Fig). Specimens EMRG-Chond-T-11 and EMRG-Chond-T-29 are fully mineralized, with the pulp cavity being completely filled with osteodentine and a sharp contact is present between the two dentine types (Fig 8G–8I, S1 and S2 Figs). A specimen representing an upper anterior tooth (EMRG-Chond-T-12) also has a hollow pulp cavity, which is half filled with osteodentine extending apically, leaving a small cavity along the inner edge of the orthodentine layer and, therefore, does not represent a fully mineralized tooth (Fig 8D–8F). The lower anterior tooth (EMRG-Chond-T-10) is fully mineralized with its pulp cavity completely filled with osteodentine (Fig 8G–8I). Not fully developed teeth of †*Hemipristis serra* with hollow pulp cavities have a more developed root and a thicker layer of orthodentine than teeth with hollow pulp cavities in *Hemipristis elongata*. It is therefore evident that the dentition of †*Hemipristis serra* shows a histology similar to that of *Hemipristis elongata*, but the mineralization sequence differs between species.

**Fig 10:**
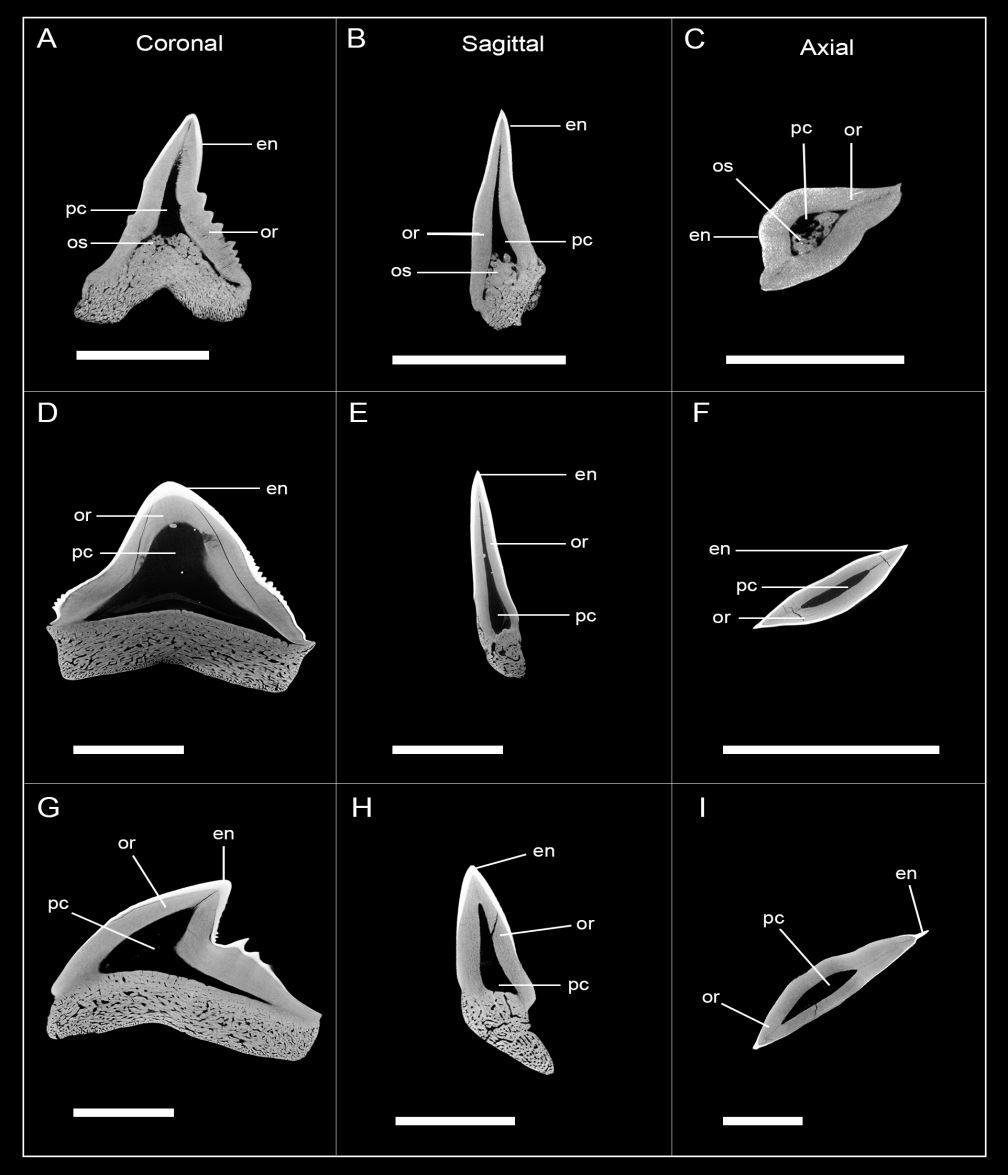
Micro-CT images of tooth sections of †*Hemipristis curvatus*, *Carcharhinus leucas*, *and Galeocerdo cuvier* in frontal, sagittal, and axial view. (A), (B), (C) †*Hemipristis curvatus* (EMRG-Chond-T-20), the pulp cavity is hollow with little intrusion of osteodentine from the root; (D), (E), (F) *Carcharhinus leucas* (EMRG-Chond-T-15), tooth with hollow pulp cavity and a shark border between the osteodentine of the root and the pulp cavity; (G), (H), (I) *Galeocerdo cuvier* (EMRG-Chond-T-16), tooth with hollow pulp cavity and a sharp border between the osteodentine of the root and the pulp cavity; scalebar = 1cm; en, enameloid; mc, Meckel’s cartilage; or, orthodentine; os, osteodentine; pc, pulp cavity; pq, palatoquadrate.

Of the ten teeth of †*Hemipristis curvatus*, two are lower anteriors, three lower laterals and five are upper laterals (Fig 10, S3–S5 Figs). All teeth show a similar degree of mineralization with open pulp cavities, which are surrounded by a very thick and prominent orthodentine layer and a thin enameloid coat. In the lower lateral tooth (EMRG-Chond-T-20), there is no clear border between the root and the pulp cavity within the crown, but there are extensions from the root osteodentine apically into the crown. The osteodentine from the root is not filling the pulp cavity completely, which results in the preservation of an unfilled pulp cavity (Fig 10A–10C). Only one tooth (EMRG-Chond-T-32) showed a filling of its pulp cavity, but the filling is not connected to the root osteodentine and appears in a brighter colour than the surrounding dentine on the CT scan, suggesting that it is a hypermineralized deposition (e.g., minerals) that invaded the pulp cavity from outside via pores of the poorly developed root dentine during the fossilization process and, therefore, represents an artefact (S5A–S5C Fig). In contrast to †*Hemipristis curvatus*, the tiger shark *Galeocerdo cuvier* and the bull shark *Carcharhinus leucas*, both having orthodont teeth, maintain a hollow pulp cavity in functional teeth (like in other carcharhiniform sharks), which is clearly separated from the root osteodentine without any intrusion from the latter into the crown (Fig 10).

### Tooth histology patterns

Although micro-CT scanning is a powerful tool to investigate tooth mineralization patterns noninvasively, the difference in density of orthodentine and osteodentine turned out to be too low to always satisfactorily resolve the presence or absence of orthodentine in an osteodont tooth. Therefore, tooth sections were prepared for better visualization of the vascular system and crystalline structures to verify the presence or absence of different dentine structures. Teeth of three species were sectioned—the bull shark *Carcharhinus leucas* (Carcharhiniformes), the shortfin mako shark *Isurus oxyrinchus* (Lamniformes), and the fossil snaggletooth shark †*Hemipristis serra* (Carcharhiniformes).

**Fig 11:**
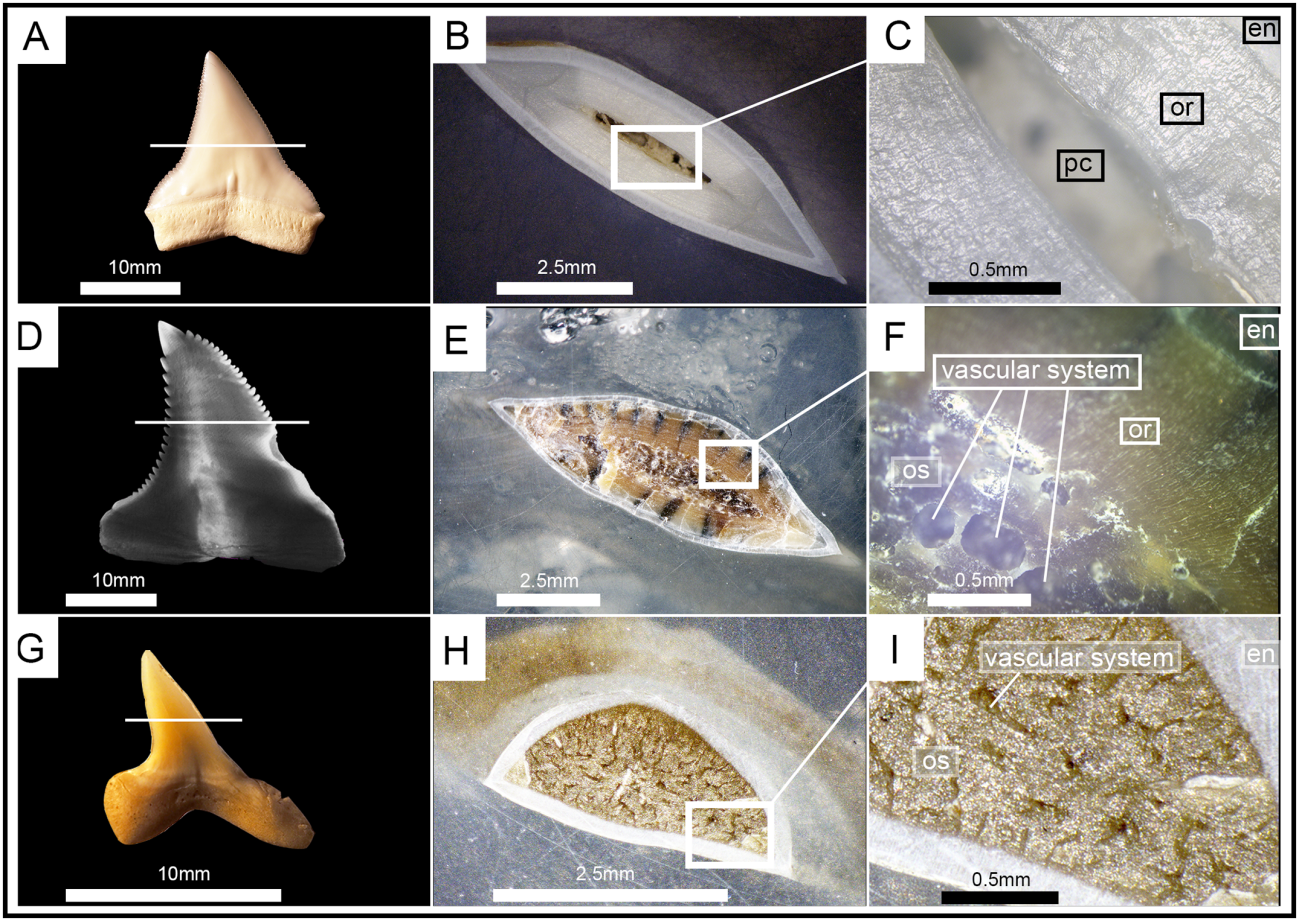
Tooth sections of an orthodont (*Carcharhinus leucas*), pseudoosteodont (†*Hemipristis serra*) and osteodont shark (*Isurus oxyrinchus*). The white line indicates the plane of the section. (A), (B), (C) *Carcharhinus leucas* (EMRG-Chond-T-15) has a hollow pulp cavity surrounded by tightly packed orthodentine; (D, E, F) fully mineralized tooth of †*Hemipristis serra* (EMRG-Chond-T-11) with a thick layer of orthodentine, surrounding the osteodentine core; (G, H, I) the crown of *Isurus oxyrinchus* (EMRG-Chond-T-14) is fully filled by osteodentine. Orthodentine is absent in this species. en, enameloid; mc, Meckel’s cartilage; or, orthodentine; os, osteodentine; pc, pulp cavity; pq, palatoquadrate.

The tooth of *Carcharhinus leucas* shows the typical orthodont tooth histology. Under a thin layer of enameloid lies a thick layer of circumpulpar orthodentine surrounding the unfilled pulp cavity (Fig 11A–11C). The crystalline structure of orthodentine is characterized by tightly packed, parallel tubules, which give it a banded appearance. Growth rings are clearly recognizable.

The tooth of *Isurus oxyrinchus* shows the typical osteodont tooth histology. The tooth has a prominent enameloid layer, covering an osteodentine core. There is no hollow pulp cavity present but instead the whole crown is fully filled with osteodentine. The osteodentine core is pervaded by a vascular system, which consists of delicate osteons that are randomly distributed and are disposed in a twisting, branchial network. Orthodentine is completely absent in the tooth of *Isurus oxyrinchus* (Fig 11G–11I).

Like in *Isurus oxyrinchus*, the pulp cavity of †*Hemipristis serra* is fully filled by an osteodont core. The vascular system within the osteodentine core differs from the one found in *Isurus oxyrinchus* in that it is composed of a few large, irregularly arranged canals directed vertically from the root to the apex of the crown. The tooth histology of †*Hemipristis serra* differs further from *Isurus oxyrinchus* as a thick layer of orthodentine is surrounding the osteodont core. The tooth is covered superficially by a thin layer of enameloid (Fig 11D–11F).

## Discussion

All observed carcharhiniform sharks, except *Hemipristis elongata*, clearly are characterized by orthodont tooth histology and maintain an unfilled pulp cavity in the centre of the crown during all developmental stages of the teeth. The only dentine present in the crown is orthodentine, with osteodentine being present but confined to the root. Combining our results with those from the literature, it seems justified to assume that the orthodont tooth histology represents the common histotype for sharks of the order Carcharhiniformes with *Hemipristis elongata* being the only extant exception to date [23,25,26,27,29,30,39].

We examined the tooth development and tooth mineralization sequence in *Hemipristis elongata* using microCT images. The hollow pulp cavity within the crown is gradually filled with osteodentine, which invades the pulp cavity basally as an extension from the root-osteodentine, extending progressively apically until the pulp cavity is filled completely and replaced by an osteodont core. The CT scans indicated the presence of a second dentine within the crown, but the difference in density of ortho- and osteodentine was too low to testify that. This additional layer is far thinner relative to the tooth size than the orthodentine layer seen in other carcharhiniform sharks. Compagno [23] stated that between the osteodont core and the enameloid was an intermediate layer of circumpulpar dentine but did not further describe it. The tooth section of †*Hemipristis serra* clearly shows that this intermediate layer of dentine has the same crystalline structure as the orthodentine in the bull shark, and therefore represents orthodentine (Fig 11); in this species it is relatively thicker than in *Hemipristis elongata*. This is also in line with previous studies, in which the circumpulpar dentine in *Hemipristis elongata* was described as orthodentine [29]. The presence of osteodentine in the crown and the absence of a hollow pulp cavity in teeth of *Hemipristis elongata* would imply that *Hemipristis elongata* indeed has an osteodont dentition and therefore shares the same tooth histology with lamniform sharks [24,25,33]. However, we strongly disagree with this interpretation, as it is evident from our study, that the tooth histology of *Hemipristis* differs from the one found in *Isurus oxyrinchus*. Two striking differences are the distinct vascularization pattern and the presence of orthodentine in *Hemipristis* (the orthodentine being absent in lamniform sharks [24,25,33]). According to this pattern we hypothesize that the tooth histology in *Hemipristis* derived from an ancestral orthodont histotype as exemplified in all other carcharhiniforms and displays a modified orthodonty instead of osteodonty, which is found only in lamniform sharks. We therefore suggest using the term osteodonty only to describe the unique tooth histology in lamniform sharks, while the combined orthodont-osteodont type reported here for *Hemipristis elongata* represents a modified type and should be referred to as pseudoosteodonty, a term which was used in the past to describe a formerly orthodont tooth, in which the pulp cavity is filled secondarily with osteodentine [27,40].

Our results illustrate the secondary replacement of the hollow pulp cavity by an osteodont core also in the fossil snaggletooth shark, †*Hemipristis serra* for the first time. Teeth of †*Hemipristis serra* have been described previously as having an unfilled pulp cavity and therefore are orthodont [23,41]. This resulted in the hypothesis that a conversion of the tooth histology within *Hemipristis* occurred during its recent evolutionary history, which resulted in an osteodont tooth histotype in the extant species *Hemipristis elongata* [23]. The observation of a single upper tooth of †*Hemipristis serra*, which displayed indications of osteodentine intrusion into the pulp cavity further reinforced the interpretation of histology conversion between both species [23]. However, our study unambiguously demonstrates that the teeth of the extinct †*Hemipristis serra* display the same histotype as the extant species with fully filled pulp cavities. We also found specimens with hollow or only half-filled pulp cavities, but this is easily explained by looking at the tooth mineralization pattern within any given row in the living *Hemipristis elongata*, which exemplifies a gradual filling of the pulp cavity during development. Further, the comparison with teeth of †*Hemipristis serra* having fully filled pulp cavities revealed that the roots were not fully mineralized in specimens having hollow pulp cavities and, therefore, are not fully developed replacement teeth. Therefore, it seems that previous studies [23,41] examined replacement teeth instead of functional teeth. This isn’t uncommon since only isolated teeth of fossil species are generally available rather than whole jaws. Also, the presence of different tooth histologies of teeth occupying different tooth positions and/or having different morphologies, as reported for hybodont sharks of the genus *Lissodus* based on isolated teeth [38], seems rather unlikely and is ruled out for *Hemipristis* by our results.

The tooth histology of the Eocene †*Hemipristis curvatus* was unknown until now. No teeth of †*Hemipristis curvatus* showed a fully infilled pulp cavity but still showed a partial invagination of root osteodentine apically into the tooth crown not seen in other carcharhiniforms. The roots are similar in development to those of †*Hemipristis serra* teeth which display a hollow pulp cavity, and therefore, are also replacement teeth. The lack of fully mineralized teeth therefore might represent an artefact and we studied only replacement teeth. Presence of fully mineralized teeth can’t be ruled out and this leads us to the prediction that fully developed teeth of †*Hemipristis curvatus* share the same tooth histology with †*Hemipristis serra* and *Hemipristis elongata*. This indicates that there was no shift from an orthodont histotype to an osteodeont histotype within the genus *Hemipristis* as proposed in the past [23] and the shift in histotypes occurred simultaneously with the origination of *Hemipristis*.

It nevertheless is apparent that the tooth mineralization of *Hemipristis* has changed over time. A thick layer of orthodentine was most prominent in the oldest species, †*Hemipristis curvatus*. It was slightly reduced in †*Hemipristis serra* in the Miocene and is hardly present in the extant species *Hemipristis elongata*. In contrast, osteodentine is hardly present in the pulp cavity of †*Hemipristis curvatus*, but fully fills the pulp cavities of †*Hemipristis serra* and *Hemipristis elongata*. The high degree of root mineralization and the thick orthodentine layer in replacement teeth of †*Hemipristis serra* further indicate a continuous replacement of orthodentine by osteodentine within the genus *Hemipristis* through time.

In this study we unambiguously demonstrate that the pseudoosteodont tooth histology found in *Hemipristis elongata* differs from the osteodont type found in lamniform sharks and, therefore, indicates that the development of similar tooth histologies represent convergent evolution. Our data show that orthodentine was reduced in thickness and replaced by osteodentine in *Hemipristis* over time. This indicates that osteodentine may have a functional or structural advantage over orthodentine in teeth of this group. This hypothesis is partly supported by data of the mechanical properties of different tooth structures, showing that hardness and simultaneously elasticity is higher in osteodont than orthodont teeth [42]. However, the same study also showed that the hardness and elasticity of the enameloid was much higher than of dentine and did not differ in the examined teeth. Furthermore, the contribution of those two materials to tooth hardness is not yet completely understood. The mechanical properties of elasmobranch teeth thus are still ambiguous and, therefore, don’t allow any predictions of the impact of different tooth histologies on tooth function yet. This should be addressed in future studies to enhance our knowledge about the biomechanics in shark teeth and its implications on feeding ecology for this group.

## Conclusions

In this study we demonstrate that the osteodont histotype found in teeth of *Hemipristis elongata* differs from the osteodont type occurring in lamniform sharks with regards to vascularization and the presence of an additional dentine type, orthodentine. Therefore, we suggest to name the tooth histotype in *Hemipristis elongata* pseudoosteodonty, as it better describes its tooth histology, which represents a combined orthodont-osteodont pattern, the latter resembling the osteodont found in lamniform sharks to some extent. The teeth in lamniform sharks lack any orthodentine, a pattern not known of any other modern shark so far. We also demonstrate that pseudoosteodonty was present to varying degrees in the extinct species †*Hemipristis serra* and †*Hemipristis curvatus*, with orthodentine being successively replaced by osteodentine over time.

With these new insights, the next step is to further examine if osteodonty as exclusive pulp filling material only occurs in lamniform sharks and if all other sharks and rays assumed to have osteodont teeth have actually a pseudoosteodont tooth histology instead. If the tooth histology turns out to be unique to lamniform sharks, this could be used as a strong diagnostic feature in future studies especially in the field of palaeontology, where researchers have to work mostly with isolated teeth.

## Supporting information

S1 Table Micro-CT scan settings for the examined shark jaws and teeth. Two values in voxel size indicate different voxel sizes for the lower (first value) and upper (second value) jaw.
(XLSX)

S2 Table Tooth files, replacement teeth, functional teeth and tooth mineralization in carcharhiniform sharks. (PDF)

S3 Table Tooth mineralization sequence in *Hemipristis elongata* (EMRG-Chond-J-1). (PDF)

S1 Fig Tooth histology of †*Hemipristis serra*. (A), (B), (C) upper lateral tooth, EMRG-Chond-T-9; (D), (E), (F) upper anterior tooth, EMRG-Chond-T-12; (G), (H), (I) lower anterior tooth, EMRG-Chond-T-10; (J), (K), (L) upper lateral tooth, EMRG-Chond-T-11. Scalebar = 1cm.
(TIF)

S2 Fig Tooth histology of †*Hemipristis serra*. (A), (B), (C) upper lateral tooth, EMRG-Chond-T-29; (D), (E), (F) upper lateral tooth, EMRG-Chond-T-30; (G), (H), (I) upper lateral tooth, EMRG-Chond-T-31.
(TIF)

S3 Fig Tooth histology of †*Hemipristis curvatus*. (A), (B), (C) upper lateral tooth EMRG-Chond-T-17, (D), (E), (F) lower anterior tooth EMRG-Chond-T-18, (G), (H), (I) upper lateral tooth EMRG-Chond-T-19. Scalebar = 1cm.
(TIF)

S4 Fig Tooth histology of †*Hemipristis curvatus*. (A), (B), (C) lower lateral tooth EMRG-Chond-T-20, (D), (E), (F) upper lateral tooth EMRG-Chond-T-21, (G), (H), (I) lower anterior tooth EMRG-Chond-T-22. Scalebar = 1cm.
(TIF)

S5 Fig Tooth histology of †*Hemipristis curvatus*. (A), (B), (C) upper lateral tooth EMRG-Chond-T-32, (D), (E), (F) lower lateral tooth EMRG-Chond-T-33, (G), (H), (I) upper lateral tooth EMRG-Chond-T-34, (J), (K), (L) lower lateral tooth EMRG-Chond-T-35.
(TIF)
