# Supplementary material for: Tooth mineralization and histology patterns in extinct and extant snaggletooth sharks, Hemipristis (Carcharhiniformes, Hemigaleidae)—Evolutionary significance or ecological adaptation?
Source: PLoS One. 2018 Aug 8;13(8):e0200951. doi: 10.1371/journal.pone.0200951 (PMC6082511; doi:10.1371/journal.pone.0200951)
Supplement: S2 Table — (PDF) [file pone.0200951.s002.pdf]

Species	*Inventorynumber*	***Row***	***Histotype***	***No Teeth***	***F***	***R***
Carcharhinus melanopterus	EMRG-Chond-J-3	LMC0	orthodont	6	1	5
		LMC1	orthodont	6	1	5
		LMC2	orthodont	6	1	5
		LMC3	orthodont	6	1	5
		LMC4	orthodont	5	0	5
		LMC5	orthodont	6	1	5
		LMC6	orthodont	6	1	5
		RPC1	orthodont	6	2	4
		LPC1	orthodont	5	2	3
		LPC2	orthodont	6	1	5
		LPC3	orthodont	6	1	5
		LPC4	orthodont	6	1	5
		LPC5	orthodont	6	1	5
		LPC6	orthodont	6	0	6
Carcharhinus obscurus	EMRG-Chond-J-5	LMC1	orthodont	5	1	4
		LMC2	orthodont	5	1	4
		LMC3	orthodont	4	1	3
		LMC4	orthodont	5	1	4
		LMC5	orthodont	5	1	3
		LMC6	orthodont	5	1	4
		LPC1	orthodont	5	1	4
		LPC2	orthodont	4	1	3
		LPC3	orthodont	5	1	4
		LPC4	orthodont	4	1	3
		LPC5	orthodont	5	1	4
		LPC6	orthodont	4	1	3
Carcharhinus signatus	EMRG-Chond-J-4	RMC1	orthodont	5	2	3
		RMC2	orthodont	6	2	4
		RMC3	orthodont	5	2	3
		RMC4	orthodont	6	2	4
		RMC5	orthodont	5	2	3
		RMC6	orthodont	6	2	4
		RPC1	orthodont	4	2	2
		RPC2	orthodont	5	2	3
		RPC3	orthodont	4	1	3
		RPC4	orthodont	5	1	4
		RPC5	orthodont	5	1	4
		RPC6	orthodont	5	2	3
Chaenogaleus macrostoma	CD042	LMC1	orthodont	6	2	4
		LMC2	orthodont	7	3	4
		LMC3	orthodont	7	2	5
		LMC4	orthodont	8	3	5
		LMC5	orthodont	7	2	5
		LMC6	orthodont	7	2	5
		LPC1	orthodont	6	2	4
		LPC2	orthodont	6	2	4
		LPC3	orthodont	6	1	5

		LPC4	orthodont	5	1	4
		LPC5	orthodont	6	1	5
		LPC6	orthodont	6	1	5
Galeocerdo cuvier	EMRG-Chond-J-13	LMC1	orthodont	5	2	3
		LMC2	orthodont	4	1	3
		LMC3	orthodont	4	1	3
		LMC4	orthodont	4	1	3
		LMC5	orthodont	4	1	3
		LMC6	orthodont	4	1	3
		LPC1	orthodont	4	1	3
		LPC2	orthodont	4	1	3
		LPC3	orthodont	5	1	4
		LPC4	orthodont	4	1	3
		LPC5	orthodont	5	1	4
		LPC6	orthodont	4	1	3
Hemigaleus microstoma	CD043	LMC1	orthodont	6	2	4
		LMC2	orthodont	6	1	5
		LMC3	orthodont	6	2	4
		LMC4	orthodont	6	1	5
		LMC5	orthodont	6	2	4
		LMC6	orthodont	6	1	5
		LPC1	orthodont	5	1	4
		LPC2	orthodont	4	1	3
		LPC3	orthodont	5	1	4
		LPC4	orthodont	4	0	4
		LPC5	orthodont	5	1	4
		LPC6	orthodont	5	1	4
Hemipristis elongata	EMRG-Chond-J-1-	LMC1	osteodont	8	1	7
		LMC2	osteodont	8	2	6
		LMC3	osteodont	8	2	6
		LMC4	osteodont	7	1	6
		LMC5	osteodont	8	2	6
		LMC6	osteodont	8	1	7
		LMC7	osteodont	8	2	6
		LMC8	osteodont	8	1	7
		LMC9	osteodont	8	1	7
		LMC10	osteodont	8	1	7
		LMC11	osteodont	8	2	6
		LMC12	osteodont	7	2	5
		LMC13	osteodont	7	2	5
		LMC14	osteodont	7	2	5
		LMC15	osteodont	7	2	5
		LMC16	osteodont	6	2	4
		LMC17	osteodont	5	1	6
		LPC1	osteodont	5	1	4
		LPC2	osteodont	5	1	4
		LPC3	osteodont	6	2	4
		LPC4	osteodont	6	1	5
		LPC5	osteodont	6	2	4
		LPC6	osteodont	6	2	4

		LPC7	osteodont	7	2	5
Hemipristis elongata	EMRG-Chond-J-2	LMC1	osteodont	7	1	6
		LMC2	osteodont	8	2	6
		LMC3	osteodont	8	2	6
		LMC4	osteodont	7	2	5
		LMC5	osteodont	8	2	6
		LMC6	osteodont	7	1	6
		LMC7	osteodont	8	2	6
		LMC8	osteodont	7	2	5
		LPC1	osteodont	7	1	6
		LPC2	osteodont	7	1	6
		LPC3	osteodont	7	2	5
		LPC4	osteodont	7	2	5
		LPC5	osteodont	7	2	5
Paragaleus randalli	CD046	LMC1	orthodont	6	2	4
		LMC2	orthodont	7	3	4
		LMC3	orthodont	6	2	4
		LMC4	orthodont	6	1	5
		LMC5	orthodont	6	2	4
		LMC6	orthodont	6	1	5
		LPC1	orthodont	5	2	3
		LPC2	orthodont	5	1	4
		LPC3	orthodont	4	1	3
		LPC4	orthodont	5	1	4
		LPC5	orthodont	5	1	4
		LPC6	orthodont	4	1	3
Prionace glauca	EMRG-Chond-J-6	LMC1	orthodont	4	2	2
		LMC2	orthodont	4	1	3
		LMC3	orthodont	4	1	3
		LMC4	orthodont	4	1	3
		LPC1	orthodont	4	1	3
		LPC2	orthodont	4	1	3
		LPC3	orthodont	4	1	3
Rhizoprionodon acutus	EMRG-Chond-J-7	LMC1	orthodont	7	2	5
		LMC2	orthodont	7	2	5
		LMC3	orthodont	6	1	5
		LMC4	orthodont	7	2	5
		LMC5	orthodont	6	1	5
		LMC6	orthodont	6	1	5
		LPC1	orthodont	5	2	3
		LPC2	orthodont	5	2	3
		LPC3	orthodont	5	1	4
		LPC4	orthodont	5	2	3
		LPC5	orthodont	5	1	4
		LPC6	orthodont	6	2	4
Sphyrna zygaena	EMRG-Chond-J-8	LMC1	orthodont	5	2	3
		LMC2	orthodont	6	2	4
		LMC3	orthodont	6	1	5
		LMC4	orthodont	5	1	4
		LMC5	orthodont	5	1	4

	LMC6	orthodont	5	1	4
	LPC1	orthodont	4	1	3
	LPC2	orthodont	5	1	4
	LPC3	orthodont	5	1	4
	LPC4	orthodont	4	0	4
	LPC5	orthodont	4	1	3
	LPC6	orthodont	4	1	4
