# Supplementary material for: Tooth mineralization and histology patterns in extinct and extant snaggletooth sharks, Hemipristis (Carcharhiniformes, Hemigaleidae)—Evolutionary significance or ecological adaptation?
Source: PLoS One. 2018 Aug 8;13(8):e0200951. doi: 10.1371/journal.pone.0200951 (PMC6082511; doi:10.1371/journal.pone.0200951)
Supplement: S3 Table — (PDF) [file pone.0200951.s003.pdf]

Hemipristis elongata	*Tooth*	*Enameloid*	*Orthodentin*	*Pulp cavity*	*Root*
	**LMC3F1**	**Complete**	**Complete**	**fully mineralized**	**fully mineralized**
	**LMC3F2**	**Complete**	**Complete**	**fully mineralized**	**fully mineralized**
	**LMC3R1**	**Complete**	**Incomplete**	**cavity almost fully filled**	**almost fully mineralized**
	**LMC3R2**	**Complete**	**Incomplete**	**cavity partially filled**	**partially mineralized**
	**LMC3R3**	**Complete**	**Incomplete, thin layer**	**start of osteodentin formation**	**Root formation**
	**LMC3R4**	**Complete**	**Absent**	**Hollow**	**Absent**
	**LMC3R5**	**Incomplete**	**Absent**	**Hollow**	**Absent**
	**LMC3R6**	**Incomplete**	**Absent**	**Hollow**	**Absent**
	**LMC4F1**	**Complete**	**Complete**	**fully mineralized**	**fully mineralized**
	**LMC4R1**	**Complete**	**Complete**	**fully mineralized**	**fully mineralized**
	**LMC4R2**	**Complete**	**incomplete**	**cavity partially filled**	**partially mineralized**
	**LMC4R3**	**Complete**	**incomplete**	**cavity partially filled**	**partially mineralized**
	**LMC4R4**	**Complete**	**Absent**	**Hollow**	**root formation**
	**LMC4R5**	**Incomplete**	**Absent**	**Hollow**	**Absent**
	**LMC4R6**	**Incomplete**	**Absent**	**Hollow**	**Absent**
	**LMC5F1**	**Complete**	**Complete**	**fully mineralized**	**fully mineralized**
	**LMC5F2**	**Complete**	**Complete**	**fully mineralized**	**fully mineralized**
	**LMC5R1**	**Complete**	**incomplete**	**cavity almost fully filled**	**partially mineralized**
	**LMC5R2**	**Complete**	**incomplete**	**cavity partially filled**	**partially mineralized**
	**LMC5R3**	**Complete**	**Incomplete, thin layer**	**start of osteodentin**	**root formation**

			formation	
LMC5R4	Complete	Absent	Hollow	Absent
LMC5R5	Incomplete	Absent	Hollow	Absent
LMC5R6	Incomplete	Absent	Hollow	Absent
LMC6F1	Complete	Complete	fully mineralized	fully mineralized
LMC6R1	Complete	Complete	fully mineralized	fully mineralized
LMC6R2	Complete	incomplete	cavity partially filled	partially mineralized
LMC6R3	Complete	incomplete	cavity partially filled	partially mineralized
LMC6R4	Complete	Absent	Hollow	Root formation
LMC6R5	Complete	Absent	Hollow	Absent
LMC6R6	Incomplete	Absent	Hollow	Absent
LMC6R7	Incomplete	Absent	Hollow	Absent
LMC7F1	Complete	Complete	fully mineralized	fully mineralized
LMC7F2	Complete	Complete	fully mineralized	fully mineralized
LMC7R1	Complete	incomplete	cavity almost fully filled	partially mineralized
LMC7R2	Complete	incomplete	cavity partially filled	partially mineralized
LMC7R3	Complete	Incomplete, thin layer	start of osteodentin formation	Root formation
LMC7R4	Complete	Absent	Hollow	Absent
LMC7R5	Incomplete	Absent	Hollow	Absent
LMC7R6	Incomplete	Absent	Hollow	Absent
LMC8F1	Complete	Complete	fully mineralized	fully mineralized
LMC8R1	Complete	incomplete	cavity almost fully filled	partially mineralized
LMC8R2	Complete	incomplete	cavity partially filled	partially mineralized

LMC8R3	Complete	incomplete	cavity partially filled	partially mineralized
LMC8R4	Complete	Absent	Hollow	Root formation
LMC8R5	Complete	Absent	Hollow	Absent
LMC8R6	Incomplete	Absent	Hollow	Absent
LMC8R7	Incomplete	Absent	Hollow	Absent
LMC9F1	Complete	Complete	fully mineralized	fully mineralized
LMC9R1	Complete	Complete	cavity almost fully filled	partially mineralized
LMC9R2	Complete	incomplete	cavity partially filled	partially mineralized
LMC9R3	Complete	incomplete	start of osteodentin formation	partially mineralized
LMC9R4	Complete	incomplete	Hollow	Root formation
LMC9R5	Complete	Absent	Hollow	Absent
LMC9R6	Incomplete	Absent	Hollow	Absent
LMC9R7	Incomplete	Absent	Hollow	Absent
LMC10F1	Complete	Complete	fully mineralized	fully mineralized
LMC10R1	Complete	Complete	fully mineralized	partially mineralized
LMC10R2	Complete	Complete	cavity almost fully filled	partially mineralized
LMC10R3	Complete	incomplete	cavity partially filled	partially mineralized
LMC10R4	Complete	Incomplete, thin layer	start of osteodentin formation	Root formation
LMC10R5	Complete	Absent	Hollow	Absent
LMC10R6	Incomplete	Absent	Hollow	Absent
LMC10R7	Incomplete	Absent	Hollow	Absent
LPC1F1	Complete	Complete	fully mineralized	fully mineralized

**LPC1R1**	**Complete**	**Complete**	**cavity almost fully mineralized**	**partially mineralized**
**LPC1R2**	**Complete**	**Incomplete**	**start of osteodentin formation**	**partially mineralized**
**LPC1R3**	**Complete**	**absent**	**hollow**	**absent**
**LPC1R4**	**Complete**	**absent**	**hollow**	**absent**
**LPC2F1**	**Complete**	**Complete**	**fully mineralized**	**fully mineralized**
**LPC2R1**	**Complete**	**Complete**	**cavity almost fully mineralized**	**partially mineralized**
**LPC2R2**	**Complete**	**Incomplete**	**cavity partially filled**	**partially mineralized**
**LPC2R3**	**Complete**	**Incomplete**	**start of osteodentin formation**	**root formation**
**LPC2R4**	**Incomplete**	**absent**	**hollow**	**absent**
**LPC3F1**	**Complete**	**Complete**	**fully mineralized**	**fully mineralized**
**LPC3F2**	**Complete**	**Complete**	**fully mineralized**	**fully mineralized**
**LPC3R1**	**Complete**	**incomplete**	**partially mineralized**	**partially mineralized**
**LPC3R2**	**Complete**	**incomplete**	**start of osteodentin formation**	**root foration**
**LPC3R3**	**Complete**	**absent**	**hollow**	**absent**
**LPC3R4**	**incomplete**	**absent**	**hollow**	**absent**
**LPC4F1**	**Complete**	**Complete**	**fully mineralized**	**fully mineralized**
**LPC4R1**	**Complete**	**Complete**	**cavity almost fully filled**	**partially mineralized**
**LPC4R2**	**Complete**	**Incomplete**	**cavity partially mineralized**	**partially mineralized**
**LPC4R3**	**Complete**	**absent**	**hollow**	**absent**
**LPC4R4**	**Complete**	**absent**	**hollow**	**absent**

LPC4R5	incomplete	absent	hollow	absent
LPC5F1	Complete	Complete	fully mineralized	fully mineralized
LPC5F2	Complete	Complete	fully mineralized	fully mineralized
LPC5R1	Complete	Incomplete	cavity partially filled	partially mineralized
LPC5R2	Complete	Incomplete	start of osteodentin formation	partially mineralized
LPC5R3	Complete	absent	hollow	absent
LPC5R4	Incomplete	absent	hollow	absent
