# Supplementary figures and images for: Tooth mineralization and histology patterns in extinct and extant snaggletooth sharks, Hemipristis (Carcharhiniformes, Hemigaleidae)—Evolutionary significance or ecological adaptation?
Source: PLoS One. 2018 Aug 8;13(8):e0200951. doi: 10.1371/journal.pone.0200951 (PMC6082511; doi:10.1371/journal.pone.0200951)

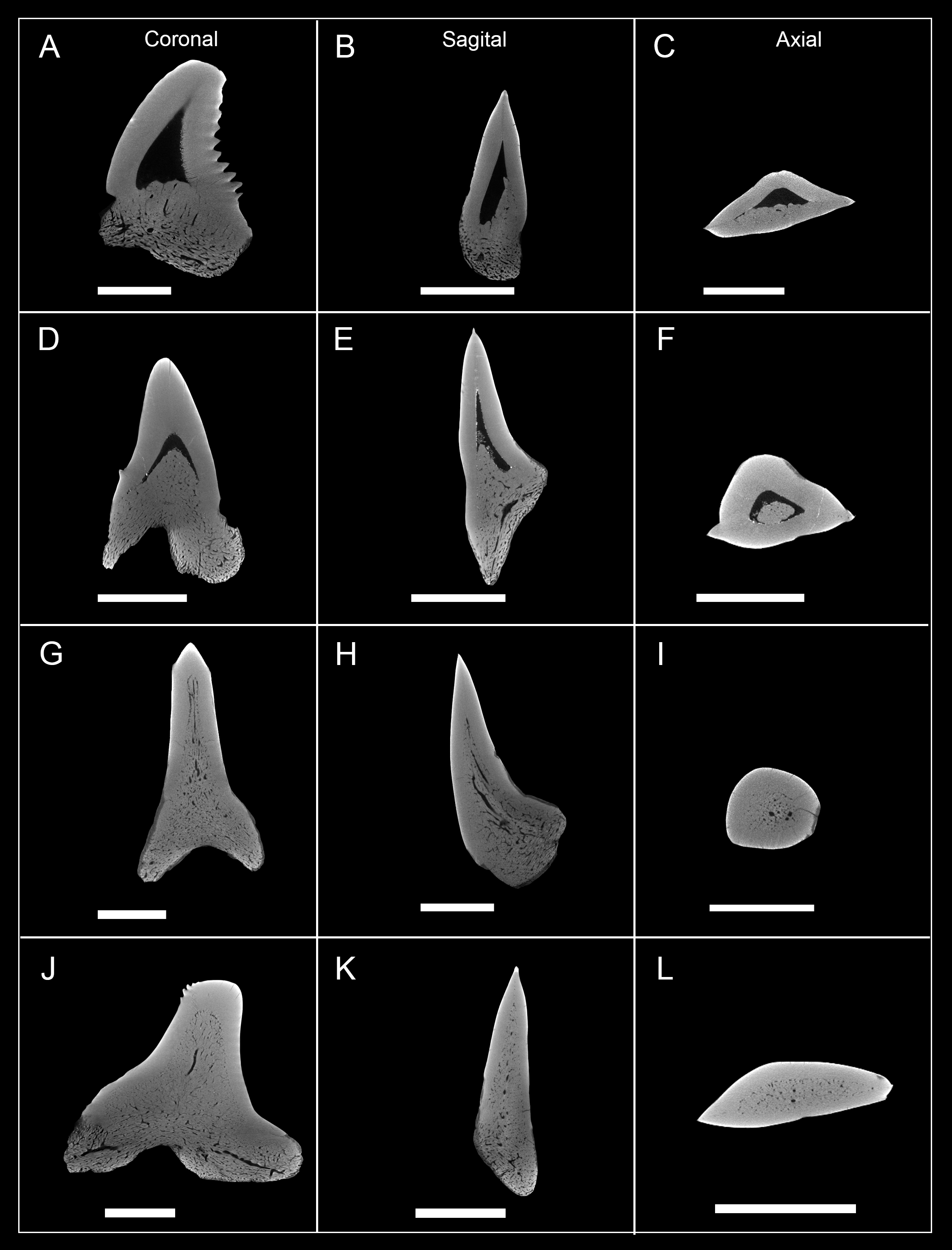

Supplement: S1 Fig — (A), (B), (C) upper lateral tooth, EMRG-Chond-T-9; (D), (E), (F) upper anterior tooth, EMRG-Chond-T-12; (G), (H), (I) lower anterior tooth, EMRG-Chond-T-10; (J), (K), (L) upper lateral tooth, EMRG-Chond-T-11. Scalebar = 1cm. (TIF) [file pone.0200951.s004.tif]

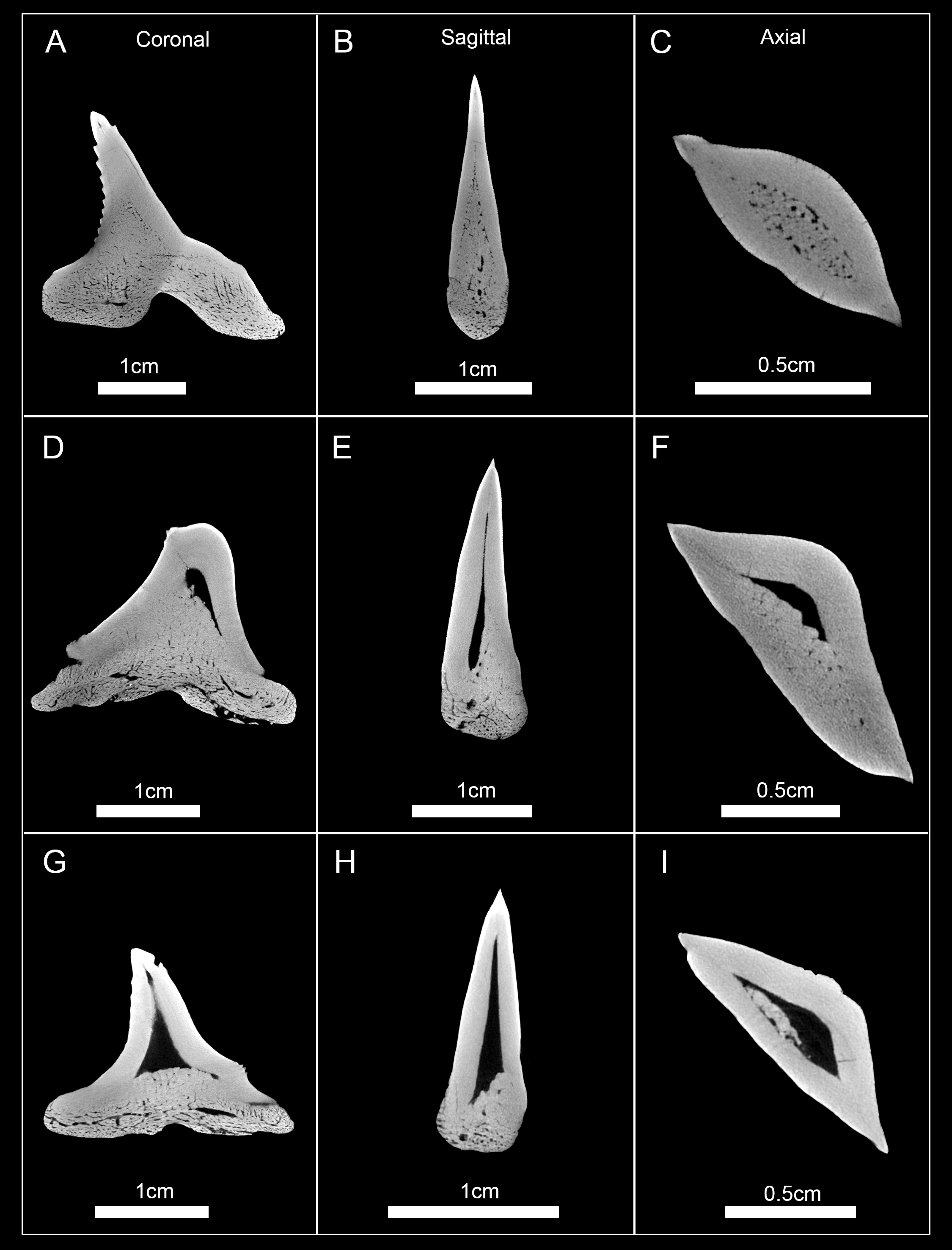

Supplement: S2 Fig — (A), (B), (C) upper lateral tooth, EMRG-Chond-T-29; (D), (E), (F) upper lateral tooth, EMRG-Chond-T-30; (G), (H), (I) upper lateral tooth, EMRG-Chond-T-31. (TIF) [file pone.0200951.s005.tif]

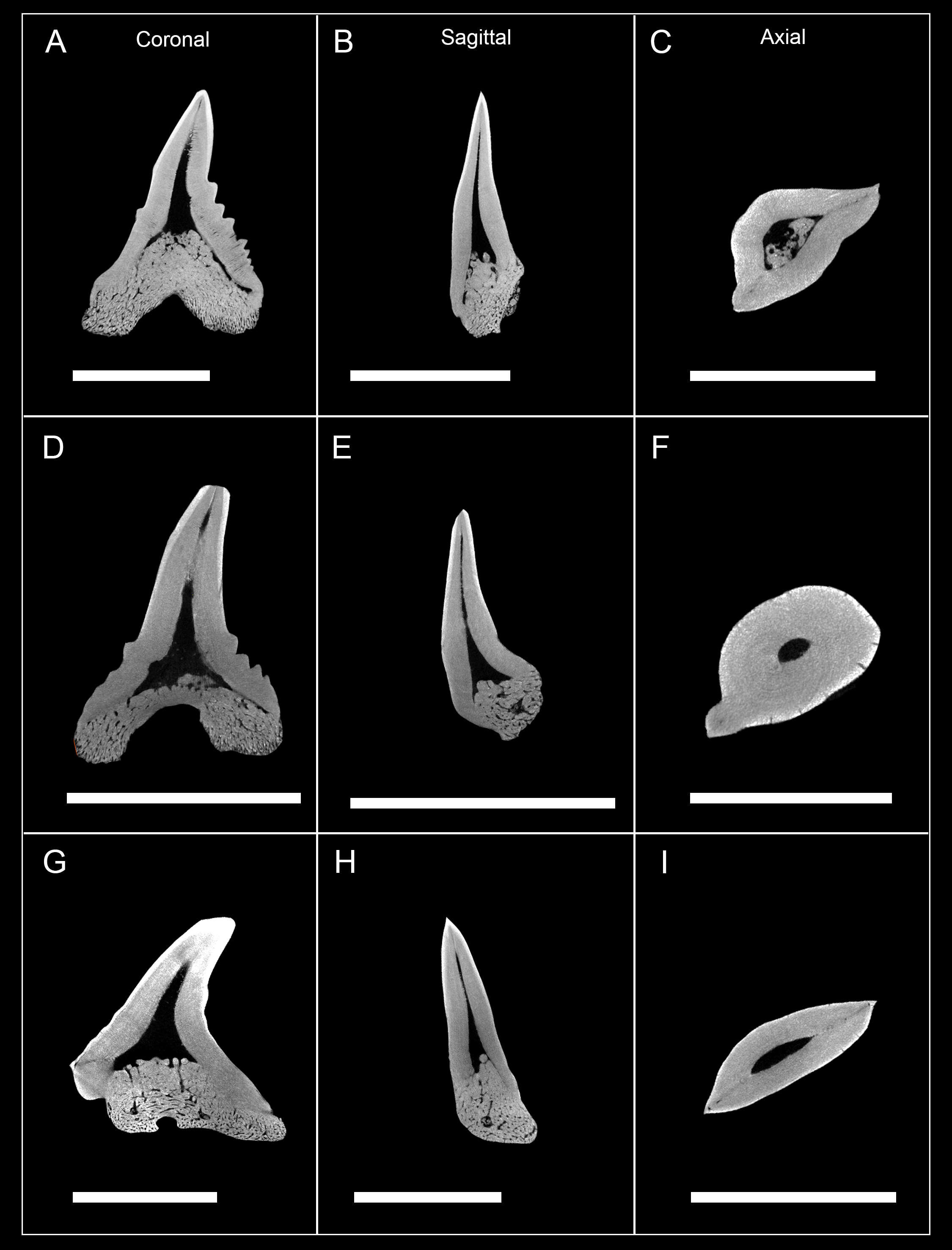

Supplement: S3 Fig — (A), (B), (C) upper lateral tooth EMRG-Chond-T-17, (D), (E), (F) lower anterior tooth EMRG-Chond-T-18, (G), (H), (I) upper lateral tooth EMRG-Chond-T-19. Scalebar = 1cm. (TIF) [file pone.0200951.s006.tif]

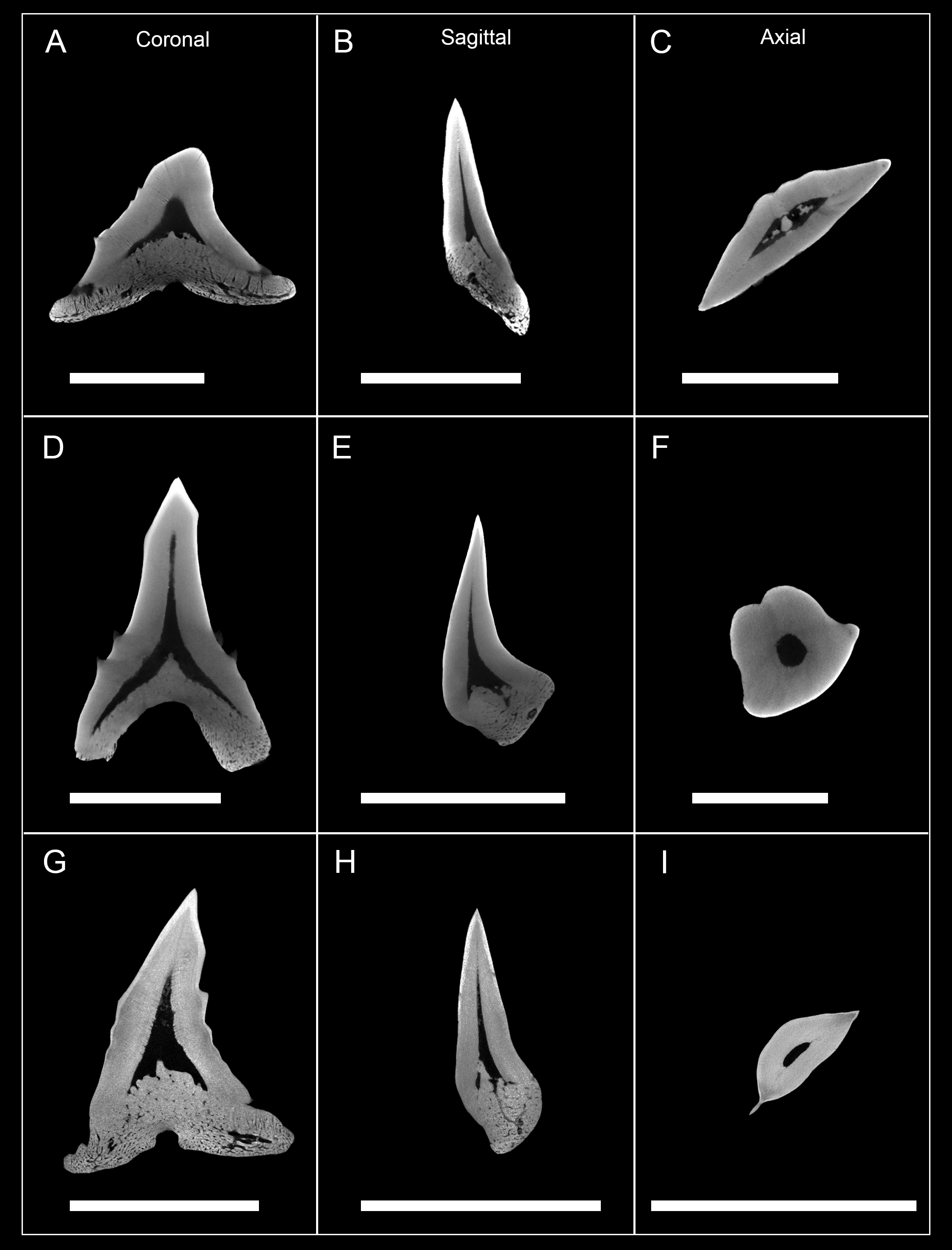

Supplement: S4 Fig — (A), (B), (C) lower lateral tooth EMRG-Chond-T-20, (D), (E), (F) upper lateral tooth EMRG-Chond-T-21, (G), (H), (I) lower anterior tooth EMRG-Chond-T-22. Scalebar = 1cm. (TIF) [file pone.0200951.s007.tif]

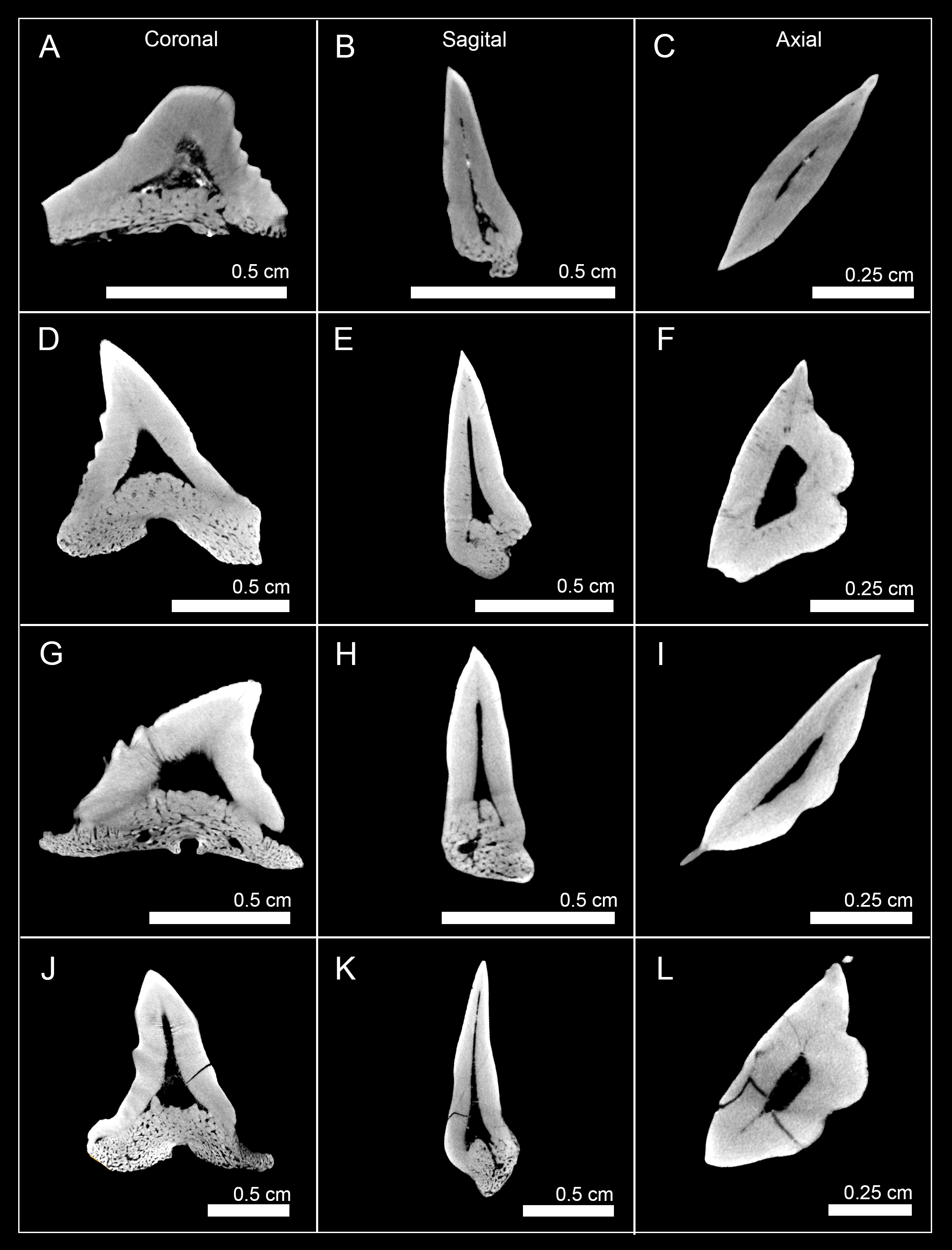

Supplement: S5 Fig — (A), (B), (C) upper lateral tooth EMRG-Chond-T-32, (D), (E), (F) lower lateral tooth EMRG-Chond-T-33, (G), (H), (I) upper lateral tooth EMRG-Chond-T-34, (J), (K), (L) lower lateral tooth EMRG-Chond-T-35. (TIF) [file pone.0200951.s008.tif]
